# Protein-protein interactions in the RPS4/RRS1 immune receptor complex

**DOI:** 10.1371/journal.ppat.1006376

**Published:** 2017-05-05

**Authors:** Sung Un Huh, Volkan Cevik, Pingtao Ding, Zane Duxbury, Yan Ma, Laurence Tomlinson, Panagiotis F. Sarris, Jonathan D. G. Jones

**Affiliations:** 1 The Sainsbury Laboratory, Norwich Research Park, Colney Lane, Norwich, United Kingdom; 2 Department of Biology and Biochemistry, University of Bath, Bath, United Kingdom; 3 Department of Biosciences, College of Life and Environmental Sciences, University of Exeter, Exeter, United Kingdom; Indiana Univ., UNITED STATES

## Abstract

Plant NLR (Nucleotide-binding domain and Leucine-rich Repeat) immune receptor proteins are encoded by *Resistance* (*R*) genes and confer specific resistance to pathogen races that carry the corresponding recognized effectors. Some NLR proteins function in pairs, forming receptor complexes for the perception of specific effectors. We show here that the Arabidopsis RPS4 and RRS1 NLR proteins are both required to make an authentic immune complex. Over-expression of RPS4 in tobacco or in Arabidopsis results in constitutive defense activation; this phenotype is suppressed in the presence of RRS1. RRS1 protein co-immunoprecipitates (co-IPs) with itself in the presence or absence of RPS4, but in contrast, RPS4 does not associate with itself in the absence of RRS1. In the presence of RRS1, RPS4 associates with defense signaling regulator EDS1 solely in the nucleus, in contrast to the extra-nuclear location found in the absence of RRS1. The AvrRps4 effector does not disrupt RPS4-EDS1 association in the presence of RRS1. In the absence of RRS1, AvrRps4 interacts with EDS1, forming nucleocytoplasmic aggregates, the formation of which is disturbed by the co-expression of PAD4 but not by SAG101. These data indicate that the study of an immune receptor protein complex in the absence of all components can result in misleading inferences, and reveals an NLR complex that dynamically interacts with the immune regulators EDS1/PAD4 or EDS1/SAG101, and with effectors, during the process by which effector recognition is converted to defense activation.

## Author summary

Paired NLR immune receptors have evolved in both plants and animals to enable host cells to detect intracellular pathogen effectors or ligands. Generally, one of the two NLR proteins in the two-component immune complex acts as a sensor that enables effector detection, while the helper (or executor) NLR activates an immune response, presumably via conformational changes. However, the mechanisms by which effector recognition activates NLR protein pairs to trigger downstream signal activation remain poorly understood. We suggest here that the Arabidopsis RPS4/RRS1 receptor forms hetero-oligomeric complexes in the nucleus both in the pre- and post-activation states. In contrast, some animal NLRs make oligomers solely upon detecting pathogen ligands. We propose that the RPS4/RRS1 pre-activation immune complex in the nucleus is converted to the activated state via changes in inter- and intra-molecular interactions upon effector recognition. RPS4/RRS1 associates with the EDS1/PAD4 complex, which is likely to be involved in downstream signaling, both before and after activation. Recognition of AvrRps4 or PopP2 by RPS4/RRS1 in association with EDS1/PAD4 does not disrupt this immune complex, but likely provokes conformational changes within it.

## Introduction

Plants and animals have evolved an effective immune system that uses both cell surface and intracellular receptors to detect pathogen invasion and then activate defense mechanisms [1–4]. Plant *Resistance* (*R*) genes mostly encode intra-cellular nucleotide-binding, leucine-rich repeat immune receptors (NLRs) that resemble similar receptors found in mammals (NLRs) [5, 6]. Most plant NLRs carry either a Toll, Interleukin-1 Receptor, Resistance protein (TIR) domain or a Coiled-coil (CC) domain at their N-termini [7, 8]. Plant NLRs directly or indirectly detect specific pathogen-derived “avirulence” (*avr*) effector proteins and activate effector-triggered immunity (ETI), which restricts the growth and spread of pathogens [9]. How plant NLR proteins activate defense upon effector recognition is poorly understood.

Plant NLRs localize to various subcellular compartments. For example, Arabidopsis *Resistance to Pseudomonas maculicola 1* (RPM1), a CC-type NLR (CNL), localizes at the plasma membrane [10]. The potato Rx protein, a typical CNL protein that confers resistance to *Potato Virus X*, shows a nucleocytoplasmic localization, and both nuclear and cytoplasmic pools are required for full defense activation [11]. Interestingly, several plant NLRs have been shown to localize to the nucleus and directly associate with transcription-regulated proteins for disease resistance activation [12–14]. For instance, *suppressor of npr1-1 constitutive 1* (SNC1), which is a TIR-NLR (TNL), localizes to both cytosol and nucleus [15]. However, SNC1 function likely requires nuclear localization because of the direct interaction between SNC1 and the transcriptional co-repressor *Topless-related 1* (TPR1). This interaction might indirectly regulate transcriptional reprogramming via *Histone deacetylase 19* (HDA19) [15, 16]. Nuclear localization of the tobacco N and *Resistance to Pseudomonas syringae 4* (RPS4) proteins is also essential for function [17, 18]. Upon effector (a viral helicase) recognition, the N protein might function in part by interactions with the transcription factor, *squamosa promoter-binding protein-like 6* (SPL6) to initiate disease resistance signaling via transcriptional reprogramming [19]. Furthermore, both SNC1 and RPS4 genetically and physically interact with *helix-loop-helix* (bHLH) type transcription factor (TF), bHLH84 [14].

The flax (*Linum usitatissimum*) L6 is a typical TNL protein that directly recognizes variants of the biotrophic flax rust fungus (*Melampsora lini*) effector AvrL567 [20]. Transient expression of the L6 TIR domain alone is sufficient for activation of defense without effector recognition [21, 22]. *RPS4* was first reported as a disease-resistance gene in Arabidopsis that specifies recognition of and response to *Pseudomonas syringae* effector AvrRps4 [23]. Furthermore, over-expression of full length *RPS4* in tobacco induces an AvrRps4-independent Hypersensitive cell death Response (HR). Similarly, RPS4 TIR domain over-expression results in AvrRps4-independent HR induction, probably via TIR-TIR self-association [24, 25]. An interface between RPS4 and *Resistance to Ralstonia solanacearum 1* (RRS1) TIR domains was revealed by X-ray crystallography [21, 25]. TIR-TIR domain interactions could play a major role in activation of cell death/resistance.

*RPS4* function requires the genetically adjacent *RRS1* gene, which encodes an atypical TNL with a C-terminal WRKY DNA binding domain [26–28]. RPS4 and RRS1 comprise a two-component plant immune receptor complex, which recognizes AvrRps4 of *P*. *syringae*, the acetyltransferase PopP2 of *Ralstonia solanacearum* and an unknown effector of *Colletotrichum higginsianum* [26, 29, 30]. Expression of the *RPS4* and *RRS1* genes is regulated by a shared promoter, which indicates that both proteins are likely to be co-expressed at comparable levels in Arabidopsis. Two distinct alleles of RRS1 have been described. The RRS1-R allele recognizes AvrRps4 and PopP2, and carries a 101 amino acid C-terminal extension after the WRKY domain. In contrast, the RRS1-S allele that recognizes AvrRps4 but not PopP2 has only an 18 amino acid C-terminal extension after the WRKY domain. Furthermore, the addition of specific C-terminal extra amino acids converts RRS1-S to RRS1-R [31]. AvrRps4 interacts with, and PopP2 acetylates, the RRS1 WRKY domain, resulting in activation of the RPS4/RRS1 complex and defense induction [31, 32]. These findings suggest that RPS4/RRS1 is a two-component immune complex in which one of the two NLR proteins has an integrated domain that enables the plant to detect effectors which target that domain, consistent with the "integrated decoy" model for the evolution of two-component immune complexes [33]. Downstream signaling upon activation of RPS4/RRS1 remains poorly understood. RPS4 TIR domain-mediated HR activation can be suppressed by co-expression with the TIR domain of RRS1 [25]. However, the autoimmune phenotype of the RRS1 auto-active mutant allele, *sensitive to low humidity 1* (*slh1*), is RPS4-dependent in Arabidopsis, as well as in *N*. *tabacum* transient assays [27, 34, 35]. Many other NLR gene pairs have been identified in both plants and animals that confer resistance to pathogens [26, 36–41].

*Enhanced disease susceptibility 1* (EDS1) encodes a lipase-homologous nucleo-cytoplasmic defense regulator protein essential for resistance conditioned by TNLs [42]. EDS1 is reported to associate with some TNL proteins such as RPS4, SNC1, and RPS6 (*Resistance* to *P*. *syringae 6*) [43]. EDS1 is functional only in conjunction with other lipase-like proteins, encoded by either *phytoalexin deficient 4* (*PAD4*), or *senescence-associated gene 101* (*SAG101*) [44]. One group reported that AvrRps4 and HopA1 effector proteins alter RPS4-EDS1 or RPS6-EDS1 association [43] and two groups reported that AvrRps4 directly interacts with EDS1, using *in vivo* co-immunoprecipitation (co-IP) and *in vitro* pull-down assays [43, 45]. It was also reported, using Bimolecular Fluorescence Complementation (BiFC) in *N*. *benthamiana* leaves and co-IP assays, that EDS1 forms cytoplasmic protein complexes with the TNL proteins RPS4 or RPS6, while the cognate bacterial effectors AvrRps4 and HopA1 disrupt these EDS1 complexes [43]. Other groups reported an inability to reproduce AvrRps4/EDS1 associations in co-IP and yeast two-hybrid (Y2H) experiments [46], perhaps indicating that any such interactions are indirect.

To attempt to resolve some paradoxes and inconsistencies in the literature regarding the nature of the RPS4/RRS1 complex and the roles of its components upon effector recognition, we used BiFC and co-IP to investigate the properties of immune complexes involving RPS4/RRS1 and EDS1/PAD4/SAG101. We found that RPS4 protein does not self-associate in the absence of RRS1, and that the previously reported RPS4 autoimmunity in tobacco and Arabidopsis is suppressed when co-expressed with RRS1. Likewise, although we could reproduce observations of RPS4 association with EDS1 in the cytoplasm in the absence of RRS1, RPS4/EDS1 association is nuclear localized when RRS1 protein is present. These data strongly emphasize the need to study RPS4 and RRS1 proteins together and not separately. Our findings suggest the existence of a nuclear-localized, complex that involves RPS4, RRS1, EDS1 and PAD4, in which these components remain present before and after recognition of AvrRps4 and PopP2 effectors. We infer that the RPS4/RRS1 immune complex undergoes dynamic intra- and inter-molecular protein-protein and domain-domain interactions to activate immune responses upon recognition of effector proteins.

## Results

### RPS4 auto-immunity is attenuated by RRS1 and RPS4 stabilization is RRS1-dependent

The oligomerization of NLRs is often required for R protein function [8]. We previously reported that the association of RRS1 and RPS4 TIR domains and their dimerization are important for defense activation and cell death signaling [25]. However, the TIR-dimerization domain mutants of RPS4 and RRS1 still co-immunoprecipitate (co-IP), which indicates that other domains contribute to this interaction.

**Fig 1:**
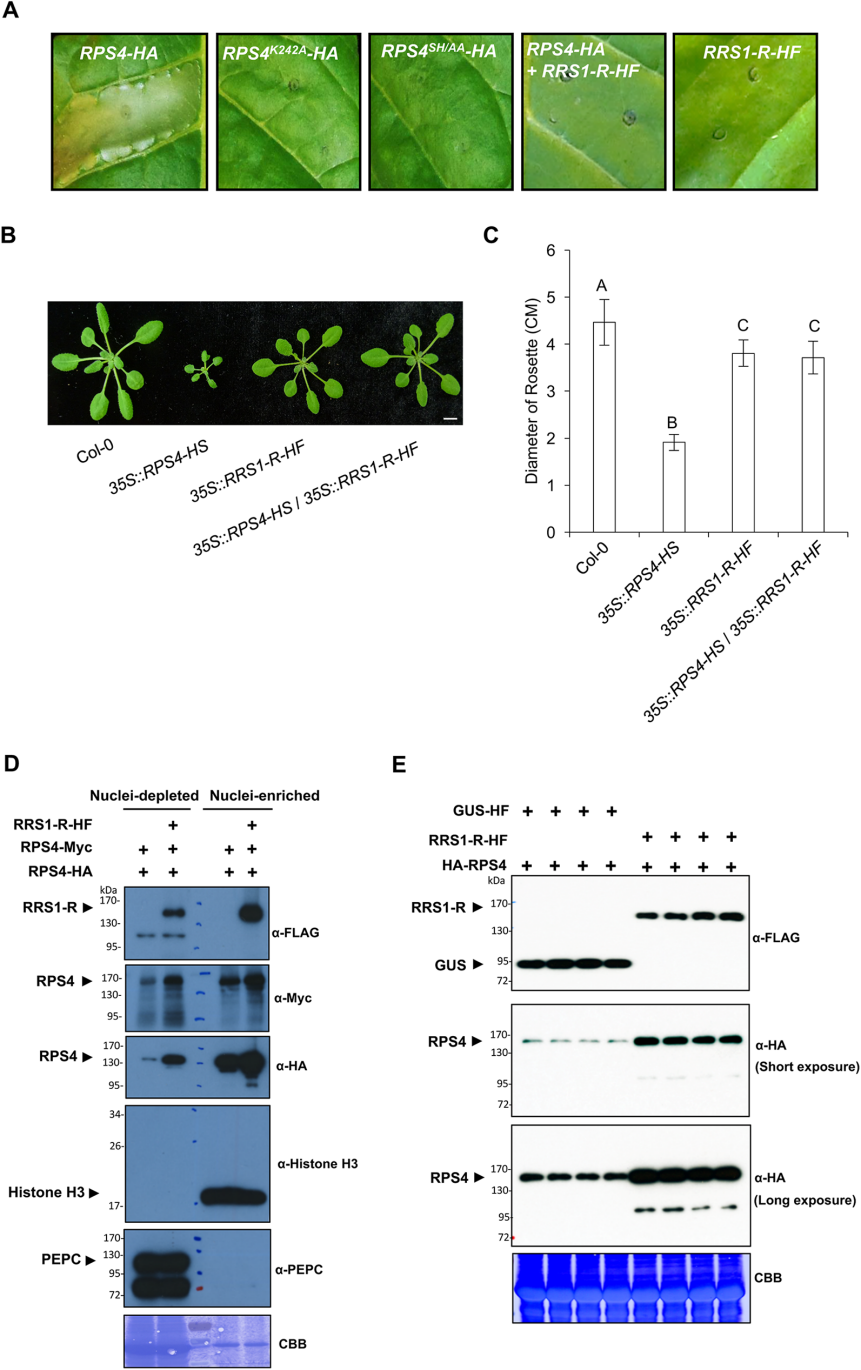
RPS4 auto-immunity is repressed by RRS1 and RRS1 increases RPS4 protein accumulation. (A) Transient overexpression of *RPS4-HA* results in auto-immunity in tobacco leaves but not *RRS1-R-HF*. The P-loop mutant (RPS4^K242A^) and the TIR-domain dimerization mutant (RPS4^SH/AA^) abolish RPS4-dependent auto-immunity. Co-expression of *RPS4-HA* with *RRS1-R-HF* blocks HR induction in tobacco leaves. (B) Stunting and dwarf phenotype of Arabidopsis transgenic line stably overexpressing *RPS4* is attenuated by crossing with *RRS1-R* transgenic Arabidopsis line. The *35S*::*RPS4-HS* / *35S*::*RRS1-R-HF* transgenic line was generated by crossing the line expressing the *35S*::*RPS4-HS* with the transgenic line with the *35S*::*RRS1-R-HF*. Images were taken with 4-week-old plants grown in short-day conditions at 22°C. Scale bar = 1.0 cm. (C) Quantification of rosette diameters at 4-week-old of the lines in (B). The leaf diameter was calculated from the plant rosette area measured in ImageJ. One-way ANOVA was used to calculate the statistical significance between genotypes, as indicated by different capital letters (P < 0.001). Bars represent mean ± SD (n = 40). (D) Fractionation of protein extracts show that RPS4 is stabilized by RRS1 in nucleus and cytoplasm. *RPS4-Myc* and *RPS4-HA* were transiently co-expressed in the presence or absence of *RRS1-HF* in *N*. *benthamiana* leaves. At 2 dpi, samples were harvested and then fractionated by the percoll-sucrose gradient method. Western blot analysis was performed with anti-FLAG, anti-Myc and anti-HA antibodies. Anti-PEPC was used as a cytosolic marker and anti-histone H3 was used as a nuclear marker. (E) RPS4-HA protein accumulation is increased by RRS1-HF expression. *RPS4-HA* and *GUS-HF* or *RPS4-HA* and *RRS1-HF* constructs in *Agrobacterium tumefaciens* were infiltrated into *N*. *benthamiana* leaves. *A*. *tumefaciens* cells were adjusted to the OD_600_ of 0.5 for *RPS4-HA* or 0.1 for *RRS1-HF* and *GUS-HF* constructs. After 2 dpi, samples were harvested and Western blots were performed using anti-FLAG and anti-HA antibodies. All the experiments were repeated three times with similar results.

Transient overexpression of RPS4 alone leads to the activation of an effector-independent HR in *N*. *tabacum* leaves [17, 24] and this autoimmune phenotype is abolished in both P-loop (RPS4^K242A^) and TIR-TIR dimerization (RPS4^SH/AA^) mutants (Fig 1A) [25]. Importantly, co-expression of *RRS1-3xHis-6xFLAG* (*HF*) with *RPS4-HA* results in abolition of the RPS4-dependent HR in tobacco leaves (Fig 1A).

Arabidopsis lines overexpressing RPS4 show constitutive defense activation giving rise to growth retardation and autoimmune phenotypes [17]. However, when such lines are crossed to *RRS1-R-HF*-overexpressing lines, their stunted phenotype is suppressed (Fig 1B and 1C) and constitutive PR1 protein accumulation is also abolished (S1A Fig). In the heteromeric RPS4/RRS1 complex, RPS4 activation only occurs upon interactions between an effector and the RRS1 WRKY domain [31, 32].

Accumulation of NLR proteins is tightly regulated, often by F-box proteins or HSP90 chaperones, and over-accumulation of many NLRs triggers an autoimmune phenotype, but the molecular mechanism of R protein complex regulation remains largely unknown [15, 17, 24, 47]. We investigated whether RRS1 protein could affect the accumulation of RPS4. We transiently co-expressed *RPS4-Myc* and *RPS4-HA* with or without *RRS1-HF* in *N*. *benthamiana* leaves. After fractionation, RPS4 protein accumulation was detected using different antibodies. Consistently, RPS4-Myc and RPS4-HA protein levels were significantly increased in the presence of RRS1 in both cytosolic and nuclear fractions (Fig 1D). To confirm this result and to check protein accumulation, we carried out Western blot analysis using HF- and HA- tagged RRS1 and RPS4 proteins, respectively. The co-expression of *RRS1* and *RPS4* leads to approximately 3.5 times more RPS4 protein, compared to the protein levels when co-expressed with *GUS* (Fig 1E). The stabilization of RPS4 protein by RRS1 was also confirmed using the *RPS4/RRS1-R* transgenic Arabidopsis plants (S1B Fig). Conceivably, reduced RPS4 accumulation in the absence of RRS1 could be due to reduced *Agrobacterium* T-DNA transfer as a consequence of the defense activation by RPS4. To test this, we evaluated GFP accumulation following transient co-expression of *35S*::*GFP* and *35S*::*RPS4* in the presence or absence of *35S*::*RRS1-R* in *N*. *benthamiana* leaves. GFP accumulation was indistinguishable in the presence or absence of RRS1 (S1C Fig).

### RRS1 enables RPS4 to co-IP with itself

We investigated localization of YFP-RPS4 in the presence of GUS-HF or RRS1-R-HF. In the absence of RRS1, the YFP-RPS4 signal is mostly seen in the nucleus, with a stronger signal in the nucleolus (S2A Fig). In the presence of RRS1-R-HF, RPS4 is also mostly seen in the nucleus but not in the nucleolus. With GUS-HF or with RRS1-R-HF, a weak YFP-RPS4 signal is also visible in the cytosol (S2B Fig).

**Fig 2:**
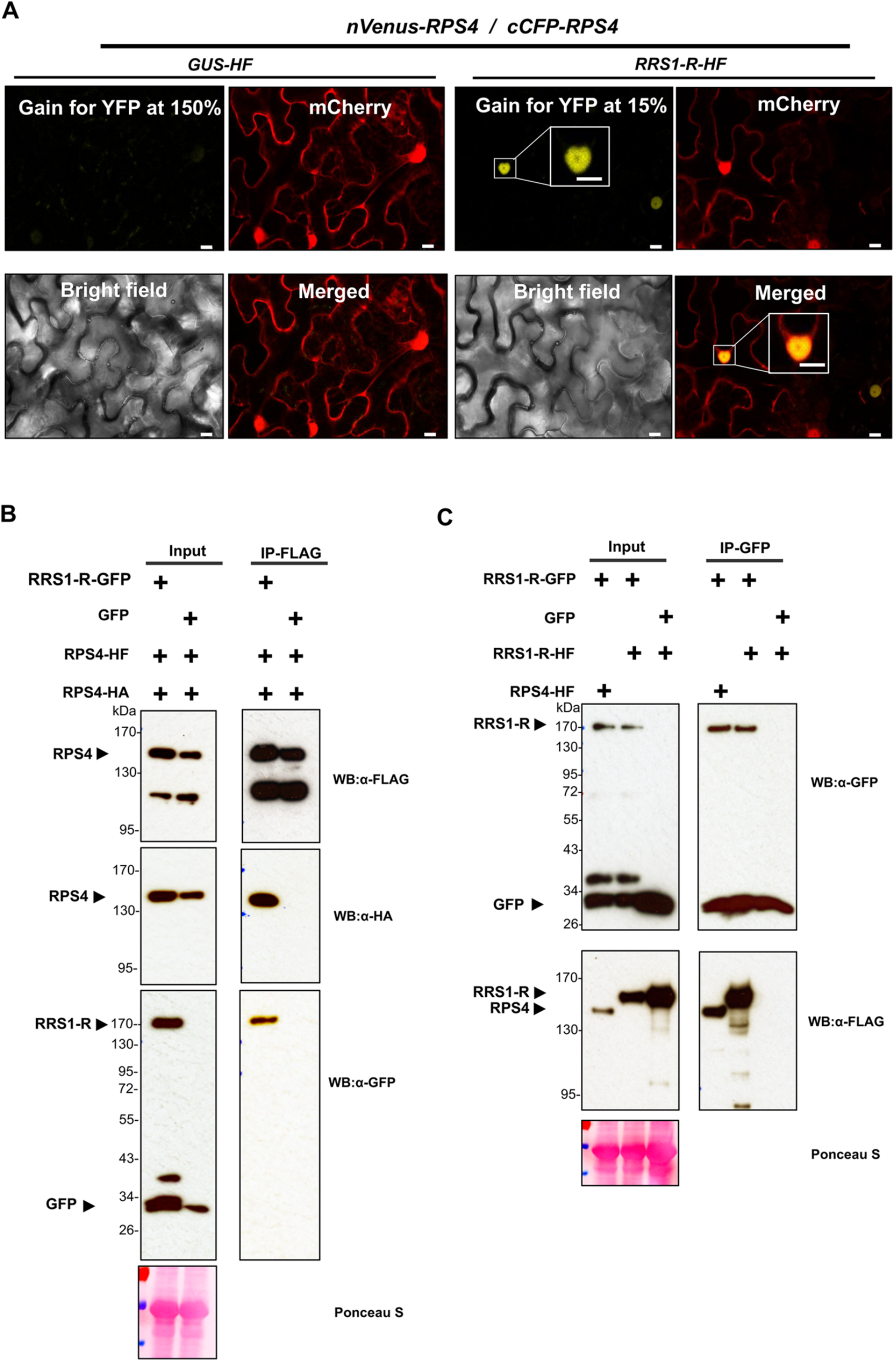
RPS4 homodimerization is dependent on RRS1. (A) BiFC assays using nVenus- and cCFP-tagged RPS4 reveal that RPS4 self-association in the nucleus is RRS1-dependent. The *nVenus-RPS4*, *cCFP-RPS4*, and *mCherry* were transiently co-expressed in the presence of *RRS1-HF* or *GUS-HF* in *N*. *benthamiana* leaves. At 2 dpi, the reconstruction YFP signal is observed with confocal microscope (Leica SP5). *mCherry* was used as a nuclear and cytoplasmic marker. Scale bar = 10 μm. (B) Co-immunoprecipitation (co-IP) assays reveal that RPS4 self-associates only in the presence of RRS1. *Agrobacterium*-mediated transient co-expression of *RRS1-GFP*/*RPS4-HF*/*RPS4-HA* or *GFP*/ *RPS4-HF*/*RPS4-HA* was performed in *N*. *benthamiana* leaves. Anti-FLAG co-IPs were performed with total protein extracts and probed with anti-GFP, -FLAG, and -HA antibodies. (C) Co-IPs show that RRS1 self-associates and forms a heteromeric complex with RPS4. Transient co-expression assays of *RRS1-GFP*/*RRS1-HF*, *RRS1-GFP*/*RPS4-HF* or *GFP*/*RRS1-HF* were performed in *N*. *benthamiana* leaves. Immunoblots show the presence of proteins in total extracts (input) and after immunoprecipitation with anti-GFP beads (IP-GFP). All the experiments were repeated at least three times with similar results.

We infer that the functional RPS4/RRS1 complex is primarily in the nucleus. Based on previous results [25, 31], we tested RPS4 and RRS1 homo- and hetero-dimeric interactions using the BiFC assay in *N*. *benthamiana*. In the absence of RRS1, no signal is observed from co-expression of cCFP-RPS4 and nVenus-RPS4 (Fig 2A). However, in the presence of RRS1, a strong RPS4 BiFC signal is seen in the nucleus. (Fig 2A). Similarly, when RPS4 is co-expressed with two different epitope tags, differently tagged RPS4 molecules co-IP with each other only in the presence of RRS1 (Fig 2B). In contrast, RRS1 can self-associate in the absence of RPS4 protein (Fig 2C). We verified these results with co-IPs using different combinations of tagged RPS4 and RRS1 proteins (S3A and S3B Fig).

### Nuclear localization of RPS4/EDS1 is enhanced by RRS1

**Fig 3:**
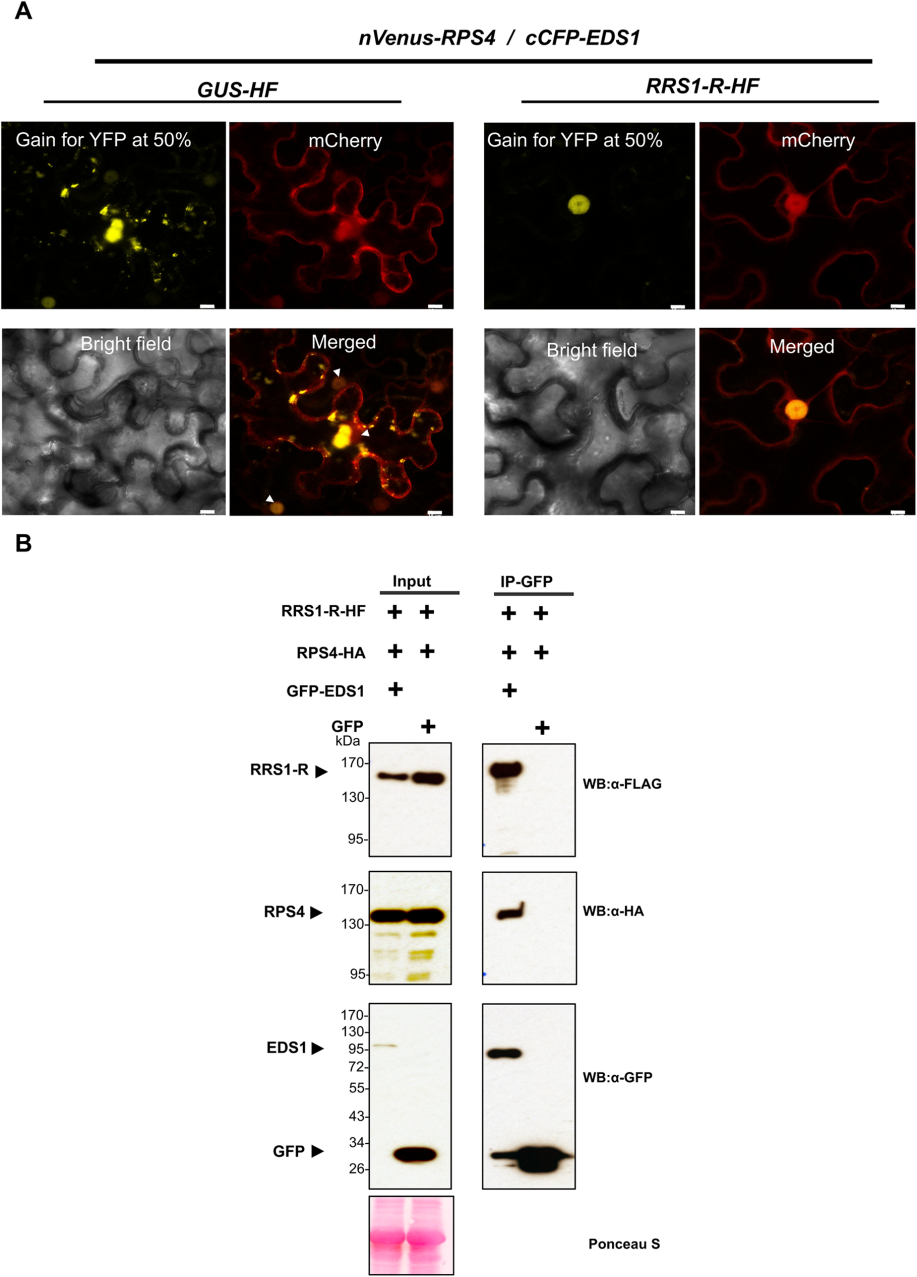
RRS1 promotes association of RPS4 and EDS1 in the nucleus. (A) In the presence of RRS1, the RPS4/EDS1 are predominantly localized to the nucleus. BiFC assays with the co-expression of *nVenus-RPS4*/*cCFP-EDS1*/*GUS-HF/mCherry* reveal reconstruction of YFP signal in the cytoplasmic aggregations and in the nucleus (arrows). In the presence of RRS1-HF, nVenus-RPS4/cCFP-EDS1 association revealed a YFP signal in the nucleus. Scale bar = 10 μm. (B) EDS1 associates with RPS4/RRS1. Upon transient co-delivery of *RPS4-HA* and *RRS1-HF* with *GFP-EDS1* or *GFP* in *N*. *benthamiana* leaves, samples were harvested at 2 dpi and total extracts were immunoprecipitated with anti-GFP beads. Specific protein-protein interactions were detected by immunoblotting with the indicated antibodies. All the experiments were repeated at least three times with similar results.

EDS1 acts as an important regulator of TNL-mediated resistance [48]. Nuclear accumulation of EDS1 is essential for TNL-mediated resistance and transcriptional activation of defense genes during ETI [42]. It has been reported that EDS1 is recruited by and physically associates with several TNL proteins such as RPS4, RPS6, and SNC1 [43]. EDS1 was reported to interact with RPS4 and other NLRs and form complexes mainly localized to punctate spots in the cytoplasm [43]. We investigated whether RRS1 could affect the cytoplasmic association of EDS1 and RPS4 [43]. To address this question, we first used BiFC assays in *N*. *benthamiana*. In the absence of RRS1, we detected reciprocal BiFC interactions of nVenus-RPS4 and cCFP-EDS1, localized to punctate spots in the cytoplasm (Fig 3A), similar to previous reports [43, 45]; this signal sometimes appeared to be adjacent to the nucleus. We also observed nuclear localization and aggregations in the cytoplasm (Fig 3A). Importantly, co-expression of *RRS1-HF* with *nVenus-RPS4*/*cCFP-EDS1* abolished the cytoplasmic signal and resulted in a nuclear-localized interaction (Fig 3A). This indicates that RRS1 is enhancing RPS4/EDS1 nuclear localization in the plant cell nucleus. Furthermore, when RPS4-HA was coexpressed with RRS1-HF and GFP-EDS1, EDS1 co-IPs with RRS1-HF, suggesting that EDS1 associates with the RPS4/RRS1 complex (Fig 3B). We transiently co-expressed *RRS1-HF* or *RPS4-HF* with *GFP-EDS1* or *GFP* in *N*. *benthamiana* leaves, and tested for co-IP. Both RRS1-HF and RPS4-HF proteins co-IP with EDS1 (S4 Fig), so the co-IP of EDS1 with RRS1 in Fig 3B could be via direct association with RRS1, and/or indirectly via association with RPS4.

### In the presence of RRS1, RPS4/EDS1 interactions are unaltered by AvrRps4

**Fig 4:**
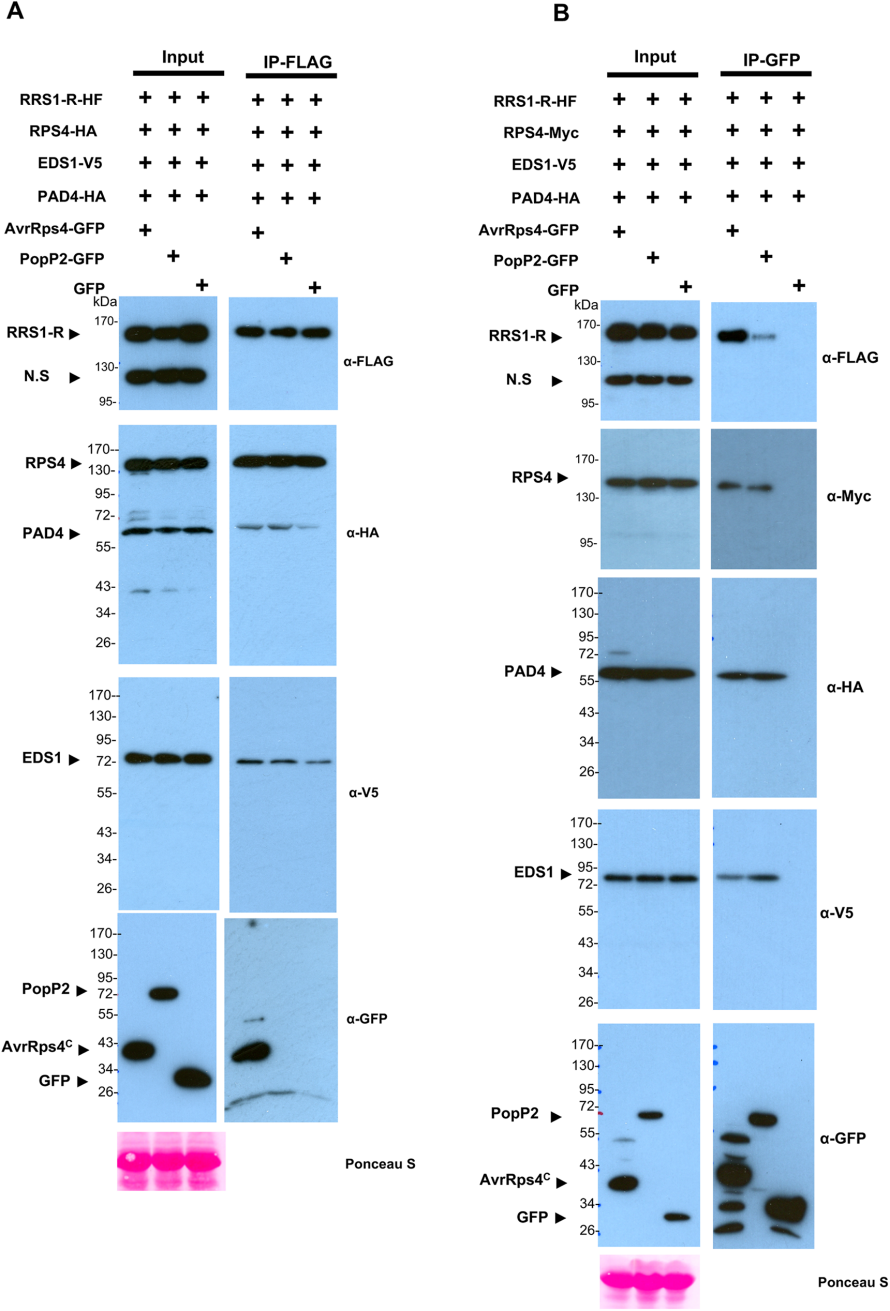
AvrRps4 and PopP2 do not disrupt the EDS1/PAD4/RPS4/RRS1 complex. (A) Anti-FLAG immunoprecipitation of RRS1-HF, RPS4, EDS1 and PAD4 in the presence and absence of AvrRps4 or PopP2. Samples were prepared from transiently co-expressed *RRS1-HF*, *RPS4-HA*, *EDS1-V5* and *PAD4-HA* in the presence of *AvrRps4-GFP*, *PopP2-GFP* or *GFP* in *N*. *benthamiana*. (B) Both AvrRps4 and PopP2 associate with RPS4/RRS1/EDS1/PAD4. To confirm effector protein association with a putative RPS4/RRS1/EDS1/PAD4 complex, samples were prepared from *N*. *benthamiana* leaves transiently co-expressing *RRS1-HF*, *RPS4-Myc*, *EDS1-V5* and *PAD4-HA* in presence of *AvrRps4-GFP*, *PopP2-GFP* or *GFP*. Total extracts were immunoprecipitated with anti-GFP beads followed by immunoblotting with the indicated antibodies. AvrRps4^C^ represents processed AvrRps4 C-terminus. All the experiments were repeated at least three times with similar results.

Both EDS1 and PAD4 are required for defense activation by the RPS4/RRS1 complex upon effector recognition [49]. Previous reports describe the disruption of RPS4/EDS1 association by the AvrRps4 and HopA1 effectors [43]. Based on this, and our findings describing the essential role of RRS1 in authentic complex formation, we investigated if RPS4/RRS1 is able to form a complex with EDS1/PAD4 *in planta* and if the RPS4/RRS1/EDS1/PAD4 complex is disrupted by AvrRps4 or PopP2. To address these questions, we co-expressed *35S*::*RRS1-HF*, *35S*::*RPS4-HA*, *35S*::*EDS1-V5*, *35S*::*PAD4-HA* with *35S*::*AvrRps4-GFP*, *35S*::*PopP2-GFP* or *35S*::*GFP* (as a negative control) in *N*. *benthamiana* leaves. Using anti-FLAG beads to select for RRS1-HF, we efficiently pulled down RPS4, EDS1 and PAD4 (Fig 4A), suggesting that EDS1/PAD4 associates with the RPS4/RRS1 complex. However, no significant disruption of this association was observed upon co-expression with AvrRps4, compared to GFP as a negative control (Fig 4A). This indicates that AvrRps4 does not affect RPS4/EDS1 association in the presence of RRS1 and PAD4. We did not observe association between PopP2 and the RPS4/RRS1/EDS1/PAD4 complex (Fig 4A) [25].

To further test this different protein/protein interactions, we co-expressed *35S*::*RRS1-HF*, *35S*::*RPS4-Myc*, *35S*::*EDS1-V5*, *35S*::*PAD4-HA* with *35S*::*AvrRps4-GFP*, or *35S*::*PopP2-GFP*, or *35S*::*GFP* in *N*. *benthamiana* and IP-ed with anti-GFP beads. Consistent with our previous observations, RPS4/EDS1 association is not disrupted by AvrRps4 in the presence of RRS1 and PAD4, while both AvrRps4 and PopP2, but not GFP, successfully pulled down all the components of the complex (Fig 4B). These findings together indicate that RPS4/RRS1 can associate with EDS1/PAD4 and this association is not disrupted by AvrRps4 or PopP2 effectors.

To further test these inferences, we used BiFC assays. We co-expressed *cCFP-EDS1*, *nVenus-RPS4*, and *RRS1-R-HF* with *AvrRps4-mCherry* or a non-functional mutant *AvrRps4*^*E187A*^*-mCherry* in *N*. *benthamiana* and observed indistinguishable YFP signals in the nucleus using the same microscope settings (S5 Fig). This suggests that AvrRps4 has no significant effect on RPS4/EDS1 association in the presence of RRS1 and these components co-localize in the nucleus.

To further verify dynamic interactions between RPS4, EDS1, and PAD4 in the presence of RRS1, we carried out multi-color BiFC analysis [50]. We co-expressed *nCerulean-RPS4*, *cCFP-EDS1*, *nVenus-PAD4*, and *RRS1-HF* with *AvrRps4-mCherry* or *AvrRps4*^*E187A*^*-mCherry*. A strong YFP signal indicating association between cCFP-EDS1 and nVenus-PAD4 is found in both the cytosol and nucleus (S6A and S6B Fig). The CFP signal was observed exclusively in the nucleus, indicating that the association between cCFP-EDS1 and nCerulean-RPS4 is mainly nuclear (S6A and S6B Fig), consistent with Fig 3A. These BiFC data suggest that localization and interaction of EDS1/PAD4 are not significantly affected by the RPS4/RRS1 complex. Furthermore, similar patterns of YFP or CFP signals in both cytosol and nucleus were observed in the presence of AvrRps4 or AvrRps4^E187A^ mutant (S6A and S6B Fig), suggesting that the RPS4/RRS1 immune complex with EDS1/PAD4 localizes mainly to the nucleus in both pre- and post-activation states.

### EDS1 interactions with AvrRps4 are blocked by PAD4 but not SAG101

**Fig 5:**
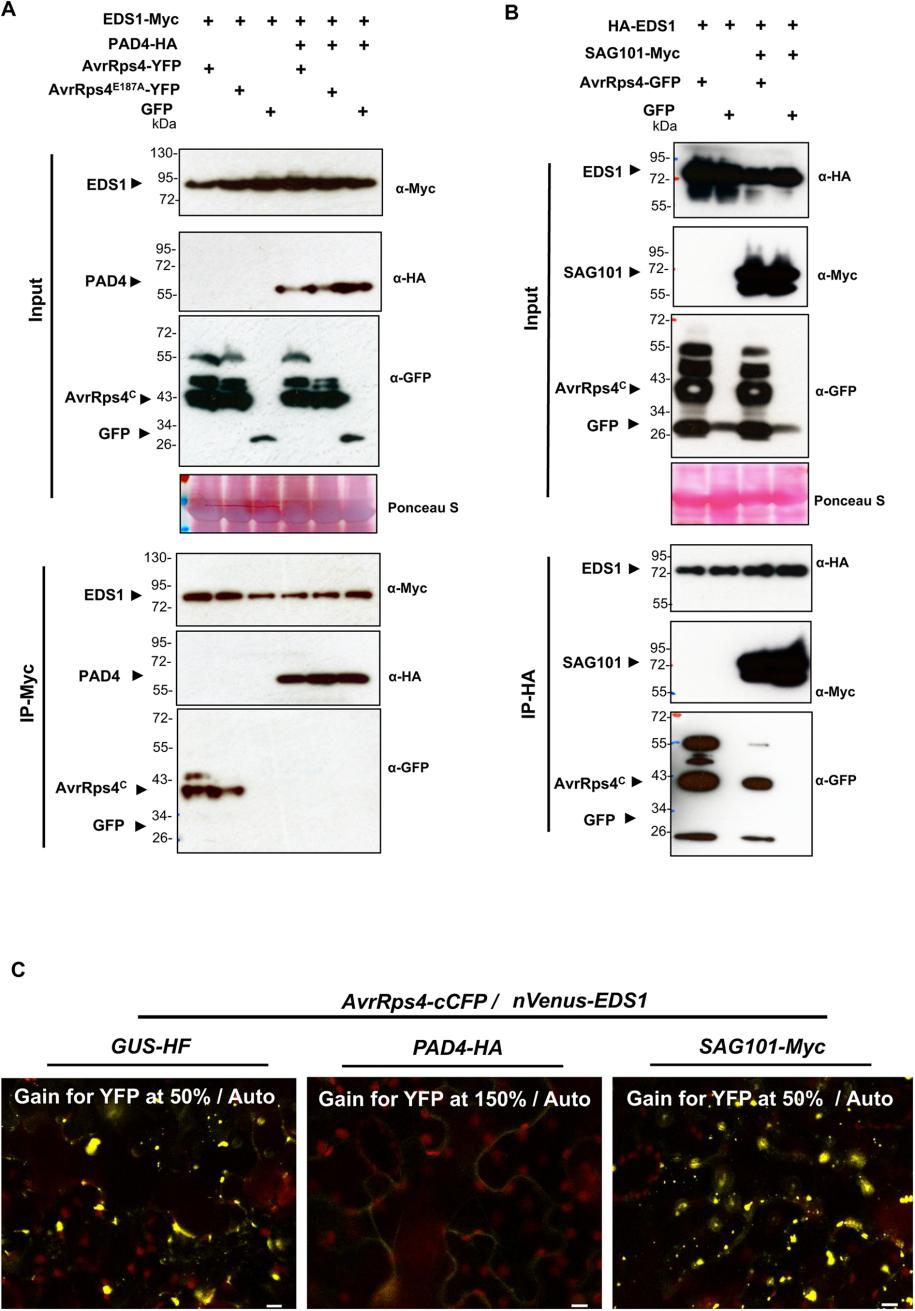
PAD4 attenuates EDS1/AvrRps4 association. (A) The EDS1/PAD4 complex strongly reduced EDS1/AvrRps4 co-immunoprecipitation *in planta*. *EDS1-Myc* or *EDS1-Myc*/PAD4*-HA* were transiently co-expressed with *AvrRps4-GFP*, *AvrRps4*^*E187A*^*-GFP* or *GFP* in *N*. *benthamiana* leaves. Immunoprecipitations were performed using anti-Myc agarose beads and then analyzed by immunoblot with the indicated antibodies. AvrRps4^C^ represents processed AvrRps4 C-terminus. (B) The EDS1/SAG101 complex can associate with AvrRps4. *HA-EDS1* or *HA-EDS1* with *SAG101-Myc* were transiently co-expressed with *AvrRps4-GFP* or *GFP* in *N*. *benthamiana* leaves. Immunoprecipitation was performed using anti-HA agarose beads and then analyzed by immunoblot with the indicated antibodies. (C) BiFC analysis reveals that EDS1/AvrRps4 interaction is reduced in the presence of PAD4-HA but not of SAG101. Cytoplasmic aggregations are reduced in the presence of PAD4. BiFC assays were performed by co-expression of the indicated proteins in *N*. *benthamiana*. Images were obtained at 2 dpi. The experiment was repeated three times with similar results. Scale bar = 10 μm.

Physical interaction between EDS1 and AvrRps4 has been reported using *in vitro* GST pull-down and co-IP assays [43, 45]. In contrast, our group previously reported no interaction between EDS1/AvrRps4 via yeast two-hybrid and co-IP assays [46]. Since EDS1 is a crucial immune signaling component, involved in several TNL-mediated defense responses [42, 48], we further examined EDS1/AvrRps4 interactions. First, we examined whether EDS1 associates with AvrRps4 *in planta*, using differentially tagged EDS1 constructs. We expressed N- or C-terminally Myc-tagged EDS1 proteins (*35S*::*Myc-EDS1* or *35S*::*EDS1-Myc*) with *35S*::*GFP* or *35S*::*AvrRps4-GFP* or *35S*::*PAD4-GFP* in *N*. *benthamiana* leaves. Following co-IP with anti-Myc beads, both AvrRps4 and PAD4 proteins could be detected with anti-GFP (S7A and S7B Fig). EDS1 associates with AvrRps4 and AvrRps4^E187A^ (Fig 5A). However, in reciprocal co-IP tests, the AvrRps4-GFP protein did not associate with EDS1-Myc or Myc-EDS1 proteins (S7A and S7B Fig), consistent with our previous data [46]. In these assays, we used PAD4 as a positive control, which strongly associates with EDS1 in both anti-GFP beads and anti-Myc IPs (S7A and S7B Fig). To further investigate EDS1/AvrRps4 interactions, we used BiFC assays. Co-expression of *AvrRps4-cCFP* with *nVenus-EDS1*, but not with *nVenus-PAD4*, gives strong nucleocytoplasmic YFP signal (S8 Fig). We also observed small aggregated foci in the cell periphery (S8 Fig). These data demonstrate that AvrRps4 can associate with the immune regulator EDS1 *in planta*.

EDS1 protein forms a heterodimeric complex with the lipase-like proteins PAD4 (in both cytosol and nucleus) and SAG101 (solely in the nucleus) [44, 51]. To test whether AvrRps4 and EDS1 can still interact in the presence of PAD4 or SAG101, we investigated the effect of PAD4 and SAG101 on AvrRps4/EDS1 association. As shown in Fig 5A, we found that PAD4 inhibits EDS1 association with both AvrRps4 and AvrRps4^E187A^, suggesting that EDS1/PAD4 hetero-dimerization might block the EDS1 surface that interacts with AvrRps4. Since PAD4 suppresses EDS1/AvrRps4 association, we then investigated whether SAG101 could also block EDS1/AvrRps4 association. We co-expressed *35S*::*HA-EDS1* and *35S*::*AvrRps4-GFP* with *35S*::*SAG101-Myc* or *35S*::*GFP* control. The co-IP results indicate that EDS1/AvrRps4 interaction was not significantly altered by SAG101 co-expression (Fig 5B).

We also investigated the effect of PAD4 and SAG101 on AvrRps4/EDS1 association using BiFC assays. We co-expressed *AvrRps4-cCFP* and *nVenus-EDS1* with *GUS* (negative control), or *PAD4-HA* or *SAG101-Myc*. Confocal microscopy indicates a specific inhibition of AvrRps4/EDS1 cytoplasmic aggregation formation by PAD4, but not by SAG101. Weak BiFC signals are detected in the cytosol and nucleus (Fig 5C). The AvrRps4^KRVY/AAAA^ inactive mutant and EDS1 also associate in the presence of SAG101 but not PAD4 in these BiFC assays (S9 Fig). These results suggest that PAD4 specifically inhibits EDS1/AvrRps4 association when transiently co-expressed in *N*. *benthamiana*.

## Discussion

Plant and animal NLRs show similar domain architectures, but do they function via similar mechanisms? In two recent studies, a paired animal immune receptor structure was investigated by cryo-electron microscopy [52, 53]. The NLR family apoptosis inhibitory proteins (NAIPs) confer pathogen perception and NLR family CARD-containing protein 4 (NLRC4) acts as an adapter to activate innate immunity via formation of an inflammasome upon bacterial ligand recognition [52, 53]. Similarly, plants carry paired NLRs. Some of these paired plant NLRs have evolved diverse ‘integrated domains’ (IDs) on one of the paired receptors. These IDs act as a sensor to detect pathogen effectors, interacting with an executor (helper) NLR to activate defense upon effector recognition [31, 33, 36, 53–55]. Conceivably, activation mechanisms of both animal and plant NLRs might involve oligomerization or homo-/heteromeric complex formation [5]. The Arabidopsis dual NLR receptor complex RPS4/RRS1 confers resistance to multiple bacterial pathogens and the fungal pathogen *C*. *higginsianum* [26, 29, 30]. Both are required for defense signaling, and form hetero-oligomers [25, 27]. Several functional studies on RPS4 without RRS1 have been reported based on the autoimmune activity of RPS4 [17, 43, 45]. In this study, we provide insight into RPS4/RRS1 protein-protein interactions in the pre-activation and post-activation states.

Several studies demonstrate the autoimmunity of RPS4 using *Agrobacterium*-based transient overexpression assays in tobacco, or stable Arabidopsis transgenic plants overexpressing *RPS4* [17, 24, 56]. It has been suggested that RPS4-mediated HR activation could be the result of homodimerization of the TIR domain, since mutations in TIR^SH/AA^ of RPS4 TIR domain or full length RPS4 prevent HR induction (Fig 1A) [24, 25]. Our findings reveal that effector-independent RPS4 autoimmunity is strongly attenuated by RRS1, both in tobacco and in Arabidopsis transgenic plants (Fig 1A and 1B). In the dual CC-NLR receptor complex comprising RGA4 and RGA5 from rice, the RGA4 autoimmunity phenotype is suppressed by RGA5 [36] indicating that sensor NLRs might act as negative regulators in multiple paired NLR systems. In addition, RRS1 overexpression in both transient tobacco system and Arabidopsis stable transgenic plants does not trigger any HR or basal defense response (Fig 1A and S1A Fig), suggesting that the primary function of the integrated domain of RRS1 is to monitor the presence of effectors. These findings indicate that RRS1 functions prior to effector perception as a negative regulator of the immune complex [56].

Homo-multimerization of RPS4 could be sufficient for cell death initiation. However, we did not see RPS4 protein homo-multimers in co-IP and BiFC experiments in *N*. *benthamiana*. RPS4-RPS4 TIR homodimerization is required to activate RPS4-mediated HR in tobacco and the RPS4-TIR domain has a self-association surface [25]. The Arabidopsis TNL protein *Recognition of Peronospora parasitica 1* (RPP1) shows self-association that involves multiple domain-domain interactions [57]. Most NLR proteins show homodimeric interactions [5], suggesting that RPS4 might also make a homodimer through TIR-TIR domain or other domain-domain interactions. Conceivably, RPS4 protein levels in our experiments are not sufficient to reveal homodimerization through co-IP or BiFC assays in the absence of RRS1 because RRS1 increases RPS4 protein accumulation. This may also indicate that a certain threshold of RPS4 protein accumulation is required for self-association, and that the act of initiating defense is associated with reduced stability of the initiating protein. RRS1 can self-associate without RPS4, and RPS4 homodimerization as a part of the RPS4/RRS1 is indistinguishable in the pre- and post-activation states. RRS1 could act as a platform that enables the correct assembly of the RPS4/RRS1 complex in the nucleus, and RPS4/RRS1 might thus form a higher-level complex comprising at least 2 RRS1 and 2 RPS4 protein molecules. RGA4/RGA5 forms a homo-/heteromeric complex in the absence of effector. However, RGA4 forms homodimers in the absence of RGA5 [36]. The behaviour of dual NLR protein complexes may vary between NLR receptors. Interestingly, effector-dependent self-association was observed in tobacco N protein upon *Tobacco mosaic virus* replicase recognition [58] and RPP1 upon *Hyaloperonospora arabidopsis* effector ATR1 recognition [56]. Other CC-type NLRs such as MLA, RPS5, and Rp1-D21 showed effector-independent self-association [5, 59, 60].

Nuclear localization and accumulation of RPS4, RRS1, and AvrRps4 are essential for an effective immune response [17, 30, 45]. Different NLR proteins are localized in various subcellular compartments in their resting states. Upon recognition of effector, some NLRs exhibit dynamic relocalization [3, 8]. It was reported that the complex between RPS4 and EDS1 mainly localizes to punctuate structures in the cytoplasm and is observed in the nucleus in steady-state or upon AvrRps4 recognition [43]. We repeated RPS4/EDS1 association assays in the presence of RRS1. Using BiFC as well as biochemical analyses, we found that in the presence of RRS1, RPS4 associates with EDS1 mainly in the nucleus (but not in the nucleolus), in both resting and activated states (Fig 3A and S6 Fig). Furthermore, based on our co-IP and BiFC data, we observed that RRS1 associates with EDS1 and RPS4, and these interactions may reflect conserved associations between TNLs and EDS1 [43].

We observed stabilization of RPS4 by RRS1 in both transient expression assays and stable transgenic Arabidopsis lines. However, using co-IP and BiFC assays, we could not find any difference in RPS4 protein accumulation between RPS4/RRS1 and RPS4/RRS1/effector combinations, suggesting that RRS1 acts as a modulator of RPS4 activity regulation via inter/intra-molecular protein-protein interactions and conformational changes.

In a previous study, it was reported that the AvrRps4 protein disrupts RPS4 association with EDS1 though interaction with EDS1, as a virulence function of AvrRps4 [43, 45] indicating that EDS1, one of the main modulator of TNL-mediate immune response, could be a target of pathogen effectors. However, these findings were obtained in the absence of RRS1, and we suggest that any such findings are misleading and do not reflect the properties or location of the authentic complex. Following these results, we investigated the disruption of RPS4/EDS1 association in the presence of RRS1 by effectors, AvrRps4 and PopP2. In both co-IP and multi-color BiFC experiments, we observed that in the presence of RRS1, AvrRps4 does not affect RPS4/EDS1 association (S5 Fig). Furthermore, RPS4, EDS1 and PAD4 continue to associate in the presence of RRS1 upon co-expression with AvrRps4 or PopP2 (Fig 4 and S6 Fig). We infer that direct interaction of AvrRps4 or PopP2 with the RRS1 WRKY domain causes RPS4/RRS1/EDS1/PAD4 complex activation, but not complex dissociation. Other host components could be, and are likely to be, associated with the RPS4/RRS1 complex in both the pre- and post- activation states.

Although we previously reported an inability to replicate this association [46], we report here that by testing more combinations of epitope tags on EDS1 and AvrRps4, we could show co-IP of these two proteins. Specifically, both N-terminally (Myc-EDS1) and C-terminally (EDS1-Myc) tagged EDS1 proteins can co-IP with AvrRps4-GFP in the IP-Myc but not IP-GFP condition (S7A and S7B Fig). We also found EDS1 and AvrRps4 associate using BiFC analysis (S8 Fig). Here our findings prove the importance of carrying out Co-IP experiments in both directions with differentially (N- or C-terminally) tagged proteins in order to avoid potential experimental artefacts. EDS1 usually makes heteromeric complexes with other lipase-like proteins, PAD4 or SAG101 in plant [44]. Importantly, we found that PAD4, but not SAG101, inhibits EDS1/AvrRps4 association, presumably via its strong affinity with EDS1 (Fig 5A and 5B). Similarly, BiFC signals observed with AvrRps4/EDS1 as cytoplasmic aggregates were specifically reduced by PAD4 co-expression but not with SAG101 (Fig 5C). Why SAG101 does not disrupt the association between AvrRps4 and EDS1, but PAD4 does, remains puzzling and requires further investigation.

Overall, our study reveals the necessity of studying proteins that are members of protein complexes in the presence of their interacting components in order to avoid misleading results. Furthermore, a significant challenge remains to address the RPS4/RRS1 conformational changes and domain/domain interactions in resting and activated states. The RPS4/RRS1 nuclear complex pre- and post-activation states are currently indistinguishable via cytology and biochemistry. Defining the dynamic changes that occur in RPS4/RRS1 upon effector recognition remains an interesting and important challenge.

## Materials and methods

### Plant materials and Agrobacterium-mediated transient transformation

*Nicotiana benthamiana* and *N*. *tabacum* plants were grown in long day conditions at 24°C [31]. Agrobacterium-mediated transient transformation assay has been described [31]. Arabidopsis plants were grown in short day conditions, at 22°C. *35S*::*RPS4-HA-StrepII (HS)* Arabidopsis transgenic plant has been described [17]. *35S*::*RRS1-R-HF* construct [31] was transformed into Arabidopsis Col-0 with the floral-dip method as described previously [61]. Homozygous *35S*::*RRS1-R-HF* plants were crossed to *35S*::*RPS4-HS* to generate double overexpression lines.

### Confocal microscopy analysis

The BiFC assay is as described previously [50, 62]. BiFC constructs using the C/N-terminal fragment of cyan fluorescent protein (cCFP) and N-terminal fragment of Cerulean (nCerulean); *nCerulean-RPS4*, *AvrRps4-cCFP*. These BiFC constructs were transformed to *Agrobacterium tumefaciens* (strain GV3101 or Agl1). Overnight cultures of *A*. *tumefaciens* cells were collected by centrifugation at 3000 rpm for 10 min. Collected cells were resuspended in Agro-infiltration buffer (10 mM MES-KOH, pH5.7 10 mM MgCl_2_). *A*. *tumefaciens* cells were adjusted to the OD_600_ of 0.5 the constructs and were transiently co-expressed in the presence of *RRS1-HF* or *GUS-HF or AvrRps4-mCheery or AvrRps4*^*E187A*^*-mCherry* constructs in *Agrobacterium tumefaciens* were infiltrated into *N*. *benthamiana* leaves. After 2 dpi, the reconstruction signals are observed with a Leica DM6000B/TCS SP5 confocal microscope (Leica Microsystems). The free mCherry is used as a nuclear/cytoplasmic marker. The experiments were repeated at least three times with similar results.

### Immuno blot and co-immunoprecipitation (co-IP) assays

Proteins were transiently expressed in 3- to 4-week-old *N*. *benthamiana* leaves and then samples were harvested at 2 dpi and ground using a mortar and pestle in liquid nitrogen. Total proteins were extracted adding cold extraction buffer [25 mM Tris-HCl, pH7.5, 150 mM NaCl, 1 mM EDTA, 10% Glycerol, 10 mM DTT, 0.2% Nonidet-40, 2% (wt/v) polyvinylpolypyrolidone, and protease inhibitor cocktail (Roche)] on ice. Samples were centrifuged at 4100 x *g* at 4°C for 25 min, and then the supernatant was filtered through two layers of Miracloth (Merck Millipore) for western blot analysis and co-IP. For Western blot, samples were boiled for 5 min with 3 x SDS sample loading buffer (25 mM Tris-HCl (pH 6.8), 300 mM DTT, 6% SDS, 0.3% bromophenol blue, and 30% glycerol). Proteins were separated by 6 or 10 or 12% SDS-PAGE, transferred to PVDF membrane (Bio-Rad) using Trans-Blot Turbo Transfer System (Bio-Rad). Immunoblot was performed with HRP-conjugated anti-HA (Roche), anti-GFP (Santa Cruz), anti-Myc (Santa Cruz), and anti-FLAG (Sigma). For co-IP, total proteins were re-centrifuged at 19000 x g at 4°C for 20 min and then the supernatant was transferred to 1.5 mL LoBind e-tube (Eppendorf). IP samples were mixed with 30 μL of anti-HA (Sigma), anti-FLAG M2 (Roche), anti-GFP (Chromotek) or anti-Myc (Santa cruz) beads and incubated at 4°C for 2 hr. Samples were washed six times with IP buffer (25 mM Tris-HCl, pH7.5, 150 mM NaCl, 1 mM EDTA, 10% Glycerol, 10 mM DTT, 0.2% Nonidet-40, and protease cocktail inhibitor). Following the final wash step, supernatant was removed using a syringe. The resin was mixed with 3 x SDS sample loading buffer and then boiled for 5 min prior to loading on SDS-PAGE gels. The experiments were repeated at least three times with similar results.

Total protein was extracted from Arabidopsis transgenic and Col-0 plants. For Western blot, proteins were separated by 10% SDS-PAGE or 16% Tris-Glycine mini protein gel (ThermoFisher). Immunoblot was performed with HRP-conjugated anti-HA (Roche), anti-FLAG (Sigma), and Pathogenesis-related protein 1 (PR1) antibody (Agrisera).

### Nuclear fractionation

Nuclear fractionation was performed using a modified protocol described by [63]. Plant tissue was ground in nuclei isolation buffer (NIB: 10 mM MES-KOH, pH 5.4, 10 mM NaCl, 10 mM KCl, 2.5 mM EDTA, 250 mM sucrose, 0.1 mM spermine, 0.5 mM spermidine, 1 mM DTT) with protease inhibitor cocktail (Roche) using a mortar and pestle. The ground tissue in NIB was filtered with Miracloth (Merck Millipore) and 10% Triton X-100 (final concentration of 0.5%) was added. The homogenate was centrifuged at 1000 x *g* for 10 min. The sucrose and Percoll layers were made by 5 ml of 2.5 M sucrose and 5 ml of 60% Percoll solution with pasteur pipette to subject the gradient to centrifugation at 1000 x g for 30 min at 4°C. Nuclei were collected from the 60% Percoll layer with a pasteur pipette and then washed with 5 volumes of NIB and 0.5% Triton X-100. After washing steps, the pellet of nuclei was resuspended with 5 ml of NIB, overlaid with 5 ml of 35% Percoll solution and centrifuged at 1000 x *g* for 10 min at 4°C. Isolated nuclear/cytosolic fractions were evaluated by western blot analysis using specific antibodies for the nuclear protein Histone H3 or the cytosolic protein phosphoenolpyruvate carboxylase (PEPC).

### Statistical analysis

Statistical analysis was carried out using the one-way analysis of variance (ANOVA).

## Supporting information

S1 Fig RPS4 protein accumulation in *Arabidopsis* and *N*. *benthamiana*. (A-B) Western blot analysis of *RPS4-HS*, *RRS1-R-HF*, and *RPS4-HS/RRS1-R-HF* transgenic lines. Total proteins were extracted from each plant and western blot was performed with anti-PR1 (A), anti-HA, and anti-FLAG (B) antibodies. (C) Reduced RPS4 accumulation is not due to reduced T-DNA transfer. *RPS4-Myc*, *GFP*, and *GUS-HF* or *RRS1-HF* constructs in *A*. *tumefaciens* were infiltrated into *N*. *benthamiana* leaves. *A*. *tumefaciens* cells were adjusted to the OD_600_ of 0.5 for *RPS4-Myc and GFP* or 0.1 for *RRS1-HF* and *GUS-HF* constructs. After 2 dpi, samples were harvested and Western blots were performed using anti-FLAG, anti-GFP, and anti-HA antibodies. All experiments were repeated three times.
(TIF)

S2 Fig RPS4 nucleolus localization is altered by RRS1 co-expression in *N*. *benthamiana*. (A) Overexpression of N-terminally YFP-tagged RPS4 with mCherry and GUS-HF results in nucleocytoplasmic localization. YFP-RPS4 mainly localizes to the nucleolus. The experiment was repeated three times with nearly identical results. Scale bar = 10 μm. (B) When co-expressing *RRS1-R-HF* and *mCherry* with *YFP-RPS4*, YFP signal is mainly observed in the nucleus but not nucleolus. Images were obtained at 2 dpi. The experiment was repeated three times with nearly identical results. Scale bar = 10 μm.
(TIF)

S3 Fig RPS4 self-associates only in the presence of RRS1 in co-IP assays. (A) *Agrobacterium*-mediated transient co-expression of *RRS1-GFP*/*RPS4-HF*/*RPS4-Myc* or *GFP*/ *RPS4-HF*/*RPS4-Myc* was performed in *N*. *benthamiana* leaves. Anti-FLAG co-IPs were performed with total protein extracts and probed with anti-GFP, -FLAG, and -Myc antibodies. (B) Co-IPs show that RRS1 self-associates and forms a heteromeric complex with RPS4. Transient co-expression assays of *RRS1-GFP*/*RRS1-HF*, *RRS1-GFP*/*RPS4-HF* or *GFP*/*RRS1-HF* were performed in *N*. *benthamiana* leaves. Immunoblots show the presence of proteins in total extracts (input) and after immunoprecipitation with anti-FLAG beads (IP-FLAG). All experiments were repeated three times.
(TIF)

S4 Fig EDS1 associates with both RPS4 and RRS1 proteins *in planta*. Co-IP was performed with transiently expressed *RRS1-R-HF* or *RPS4-HF* with *GFP-EDS1* or *GFP* in *N*. *benthamiana* leaves. After 2 dpi, samples were harvested and then immunoprecipitated with anti-GFP beads. The samples were then analyzed by immunoblotting with anti-FLAG and anti-GFP antibodies. All experiments were repeated three times.
(TIF)

S5 Fig AvrRps4 does not affect RPS4/EDS1 association in the nucleus in the presence or absence of RRS1. BiFC assays of RPS4/EDS1 association in the presence of RRS1 or both RRS1 and AvrRps4 or AvrRps4^E187A^. *N*. *benthamiana* leaves were co-infiltrated with *nVenus-RPS4*/*nCFP-EDS1*/*RRS1-R-HF/AvrRps4*^*E187A*^ or *nVenus-RPS4*/*nCFP-EDS1*/*RRS1-R-HF*/*AvrRps4-mCherry*, reconstructed YFP signals (nVenus/nCFP combination) were observed at 2 dpi. In the presence of RRS1-R-HF, both cCFP-RPS4/nCFP-EDS1/AvrRps4E187A-mCherry and cCFP-RPS4/nCFP-EDS1/AvrRps4-mCherry complex provided similar nuclear YFP fluorescence. The experiment was repeated three times. Scale bar = 15 μm.
(TIF)

S6 Fig AvrRps4 does not affect RPS4/EDS1/PAD4 association in the nucleus in the presence RRS1. (A-B) Multi-color BiFC analysis between RRS1, RPS4, EDS1 and PAD4 in the presence or absence of AvrRps4. *RRS1-HF*, *nCerulean-RPS4*, *cCFP-EDS1* and *nVenus-PAD4* were transiently co-expressed with *AvrRps4-E187A-mCherry* or *AvrRps4-mCherry*, in *N*. *benthamiana* leaves. Co-expression of *nCerulean-RPS4* and *cCFP-EDS1* resulted in the reconstitution of CFP fluorescence within the nucleus. Co-expression of *cCFP-EDS1* and *nVenus-PAD4* reconstructed YFP fluorescence in both the nucleus and cytoplasm. No significant differences were observed in the presence of AvrRps4 or AvrRps4^E187A^-mCherry for both combinations. The experiment was repeated three times with similar results. Scale bar = 15 μm.
(TIF)

S7 Fig EDS1 interacts with AvrRps4. (A-B) Both N- and C-terminally Myc tagged EDS1 co-immunoprecipitate with AvrRps4 *in planta*. The *35S*::*Myc-EDS1* or the *35S*::*EDS1-Myc* were co-infiltrated with the *35S*::*PAD4-GFP*, *35S*::*AvrRps4-GFP* or *35S*::*GFP* in *N*. *benthamiana* leaves and samples were harvested at 2 dpi. Immunoprecipitations were performed using anti-GFP and anti-Myc agarose beads. Specific protein-protein interactions were detected by immunoblotting with the indicated antibodies. AvrRps4^C^ represents processed AvrRps4C-terminus. The experiment was repeated three times with similar results.
(TIF)

S8 Fig BiFC verification of the interaction between EDS1 and AvrRps4. The *AvrRps4-cCFP* and *nVenus EDS1* constructs were transiently co-expressed in *N*. *benthamiana* leaves. The combination of *AvrRps4-cCFP* with *nVenus-PAD4* was used as a negative control. The functionality of *nVenus-PAD4* construct was verified by co-expression with *cCFP-EDS1*. Red or blue fluorescence is the indicative of chloroplast auto-fluorescence. Reconstitution of yellow fluorescence protein (YFP) indicates protein-protein interactions. The experiment was repeated three times with similar results. Scale bar = 15 μm.
(TIF)

S9 Fig AvrRps4^KRVY/AAAA^ mutant and EDS1 association in BiFC assay. BiFC reveals that interaction between of EDS1 and AvrRps4^KRVY/AAAA^ mutant forms cytoplasmic aggregations that are reduced in the presence of PAD4-HA but not in the presence of SAG101. BiFC assays were performed by co-expression of the indicated proteins in *N*. *benthamiana*. Scale bar = 15 μm.
(TIF)
