# Supplementary figures and images for: Protein-protein interactions in the RPS4/RRS1 immune receptor complex
Source: PLoS Pathog. 2017 May 5;13(5):e1006376. doi: 10.1371/journal.ppat.1006376 (PMC5435354; doi:10.1371/journal.ppat.1006376)

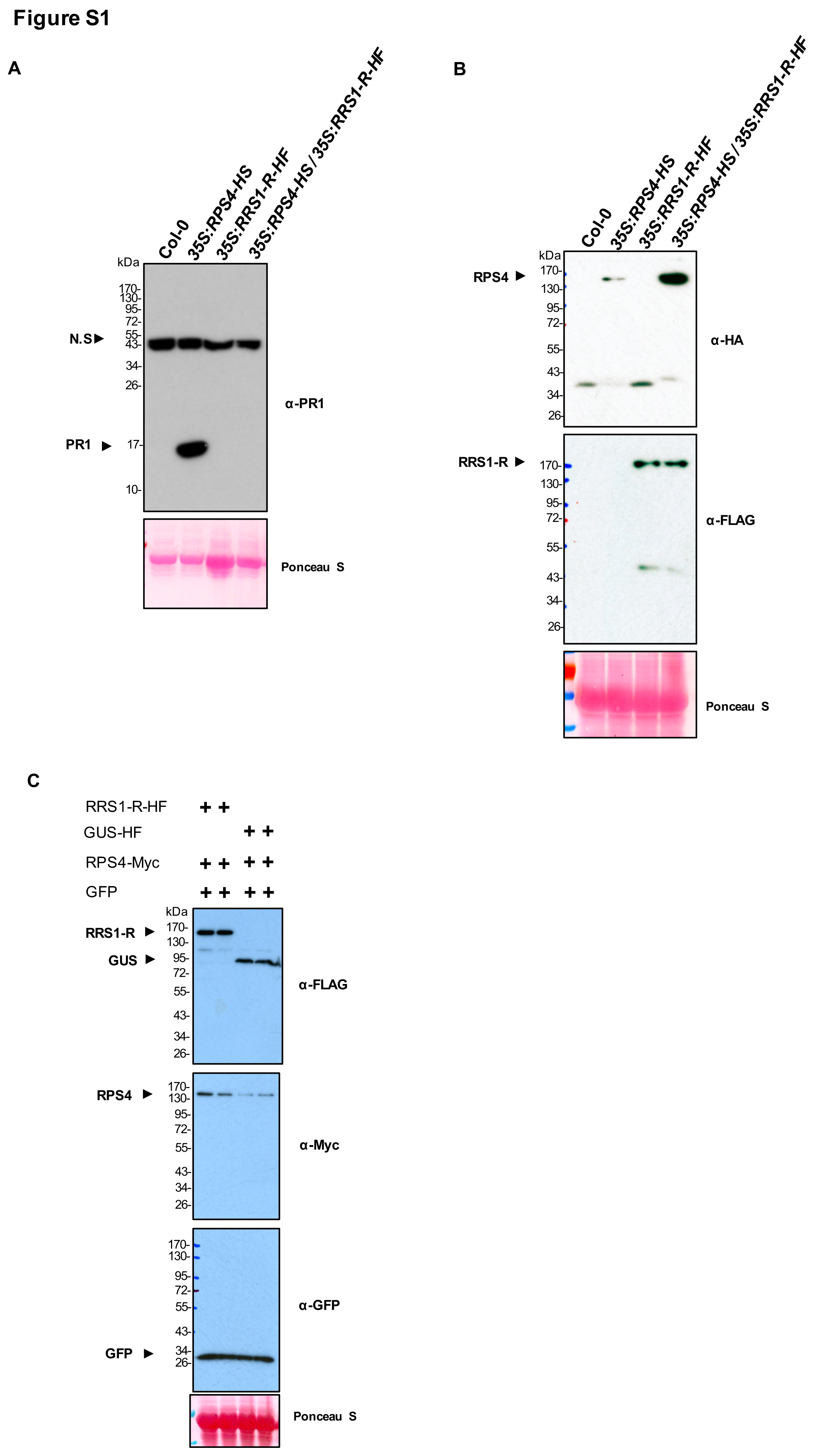

Supplement: S1 Fig — (A-B) Western blot analysis of RPS4-HS, RRS1-R-HF, and RPS4-HS/RRS1-R-HF transgenic lines. Total proteins were extracted from each plant and western blot was performed with anti-PR1 (A), anti-HA, and anti-FLAG (B) antibodies. (C) Reduced RPS4 accumulation is not due to reduced T-DNA transfer. RPS4-Myc, GFP, and GUS-HF or RRS1-HF constructs in A. tumefaciens were infiltrated into N. benthamiana leaves. A. tumefaciens cells were adjusted to the OD600 of 0.5 for RPS4-Myc and GFP or 0.1 for RRS1-HF and GUS-HF constructs. After 2 dpi, samples were harvested and Western blots were performed using anti-FLAG, anti-GFP, and anti-HA antibodies. All experiments were repeated three times. (TIF) [file ppat.1006376.s001.tif]

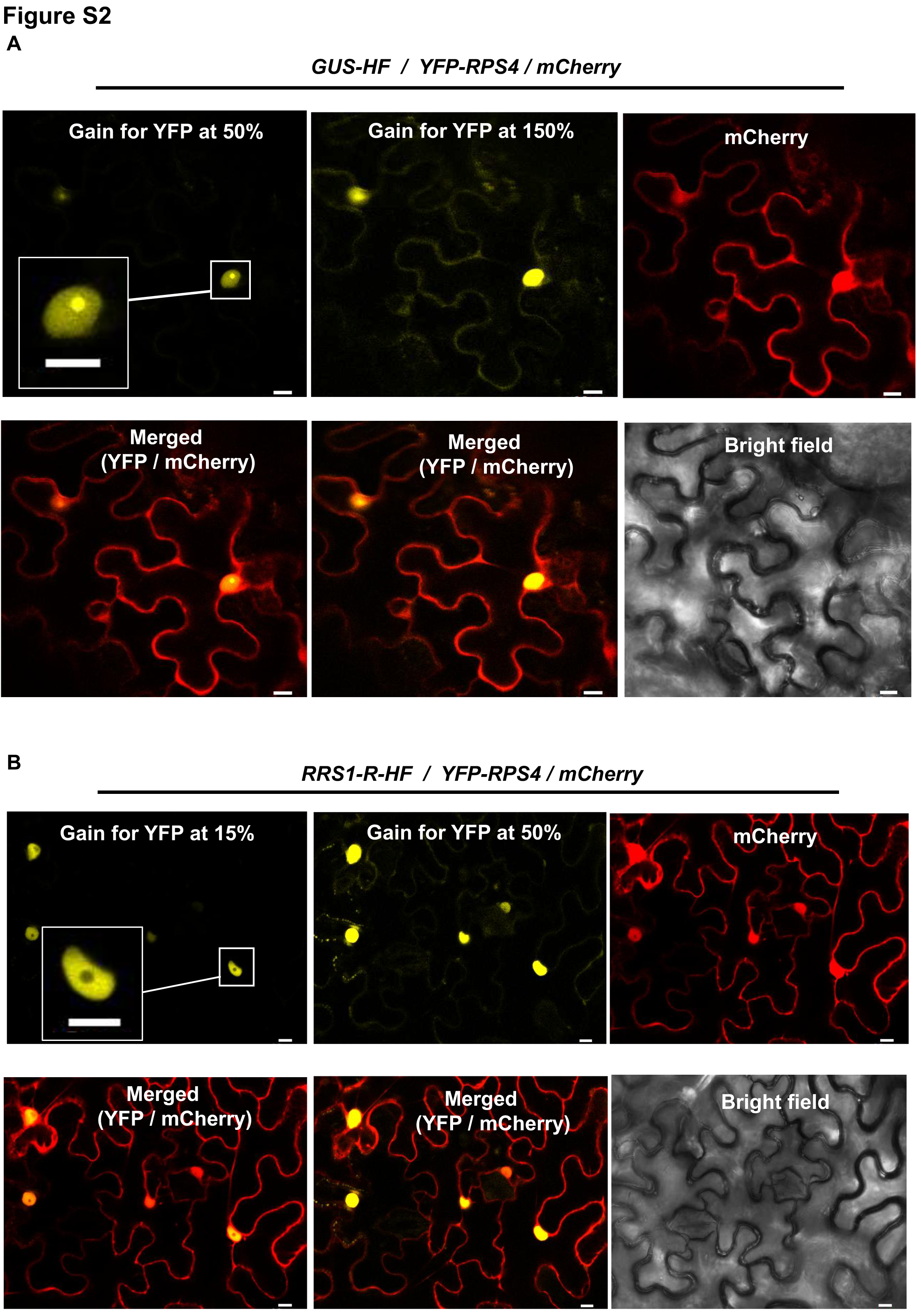

Supplement: S2 Fig — (A) Overexpression of N-terminally YFP-tagged RPS4 with mCherry and GUS-HF results in nucleocytoplasmic localization. YFP-RPS4 mainly localizes to the nucleolus. The experiment was repeated three times with nearly identical results. Scale bar = 10 μm. (B) When co-expressing RRS1-R-HF and mCherry with YFP-RPS4, YFP signal is mainly observed in the nucleus but not nucleolus. Images were obtained at 2 dpi. The experiment was repeated three times with nearly identical results. Scale bar = 10 μm. (TIF) [file ppat.1006376.s002.tif]

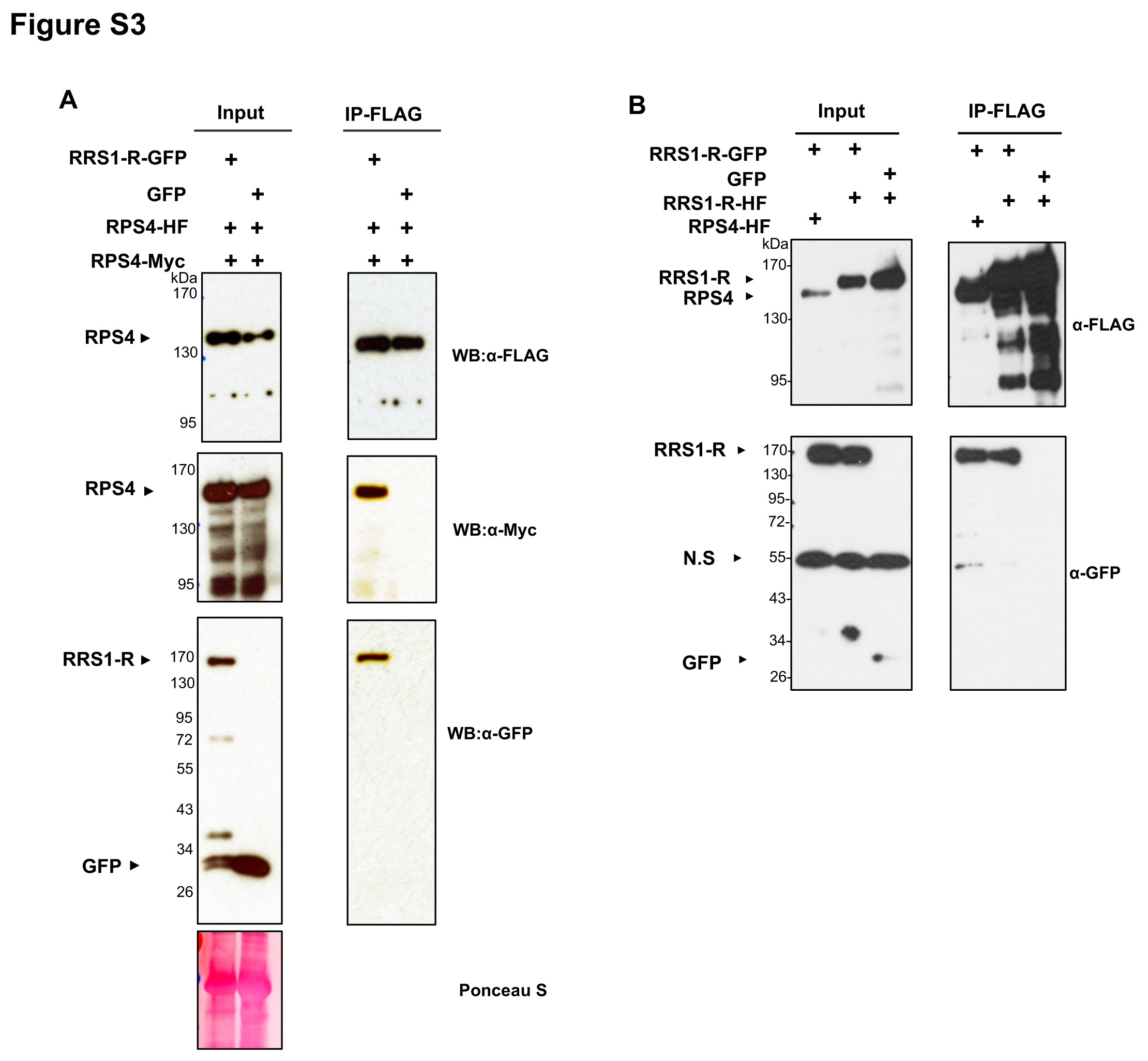

Supplement: S3 Fig — (A) Agrobacterium-mediated transient co-expression of RRS1-GFP/RPS4-HF/RPS4-Myc or GFP/ RPS4-HF/RPS4-Myc was performed in N. benthamiana leaves. Anti-FLAG co-IPs were performed with total protein extracts and probed with anti-GFP, -FLAG, and -Myc antibodies. (B) Co-IPs show that RRS1 self-associates and forms a heteromeric complex with RPS4. Transient co-expression assays of RRS1-GFP/RRS1-HF, RRS1-GFP/RPS4-HF or GFP/RRS1-HF were performed in N. benthamiana leaves. Immunoblots show the presence of proteins in total extracts (input) and after immunoprecipitation with anti-FLAG beads (IP-FLAG). All experiments were repeated three times. (TIF) [file ppat.1006376.s003.tif]

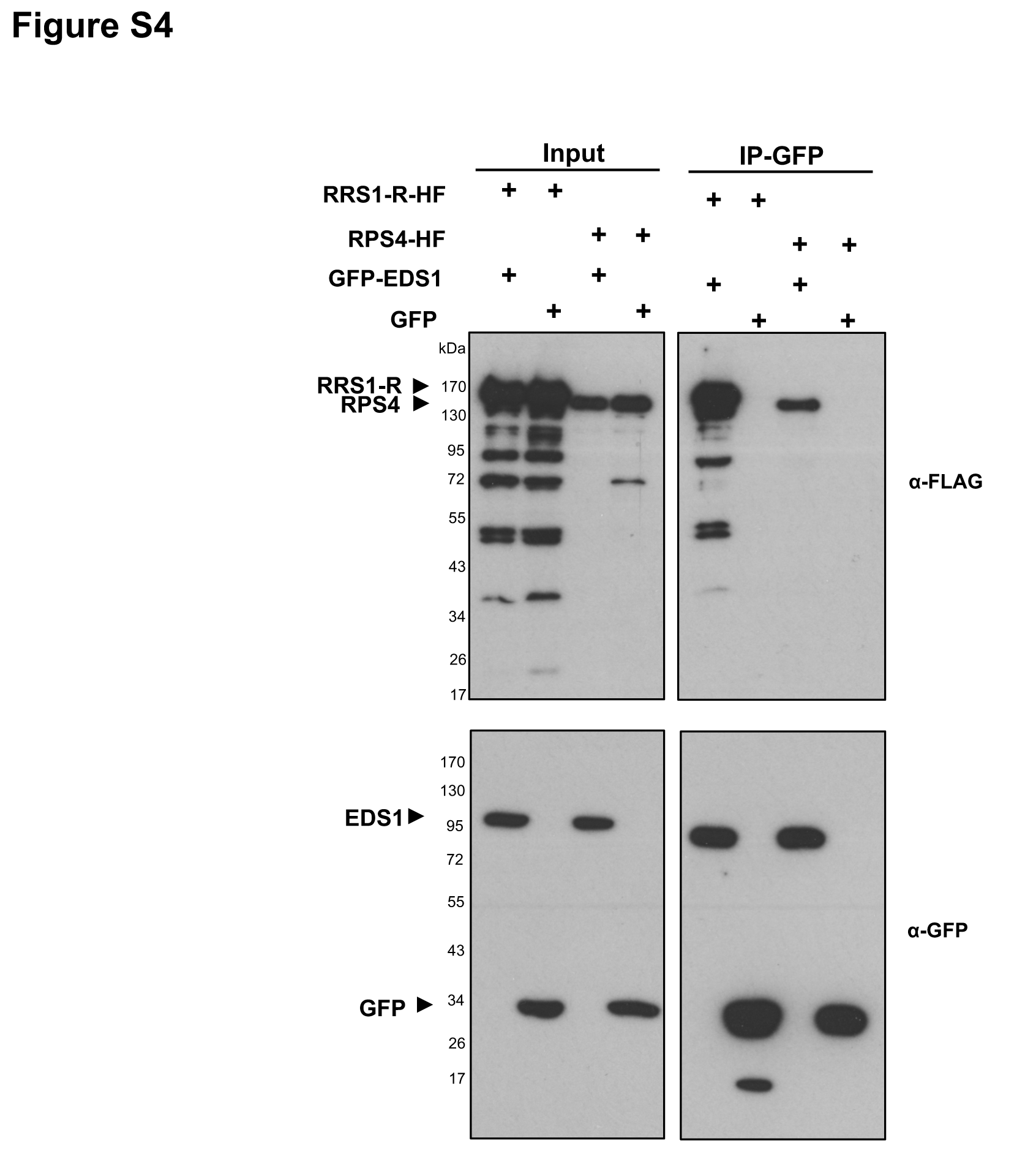

Supplement: S4 Fig — Co-IP was performed with transiently expressed RRS1-R-HF or RPS4-HF with GFP-EDS1 or GFP in N. benthamiana leaves. After 2 dpi, samples were harvested and then immunoprecipitated with anti-GFP beads. The samples were then analyzed by immunoblotting with anti-FLAG and anti-GFP antibodies. All experiments were repeated three times. (TIF) [file ppat.1006376.s004.tif]

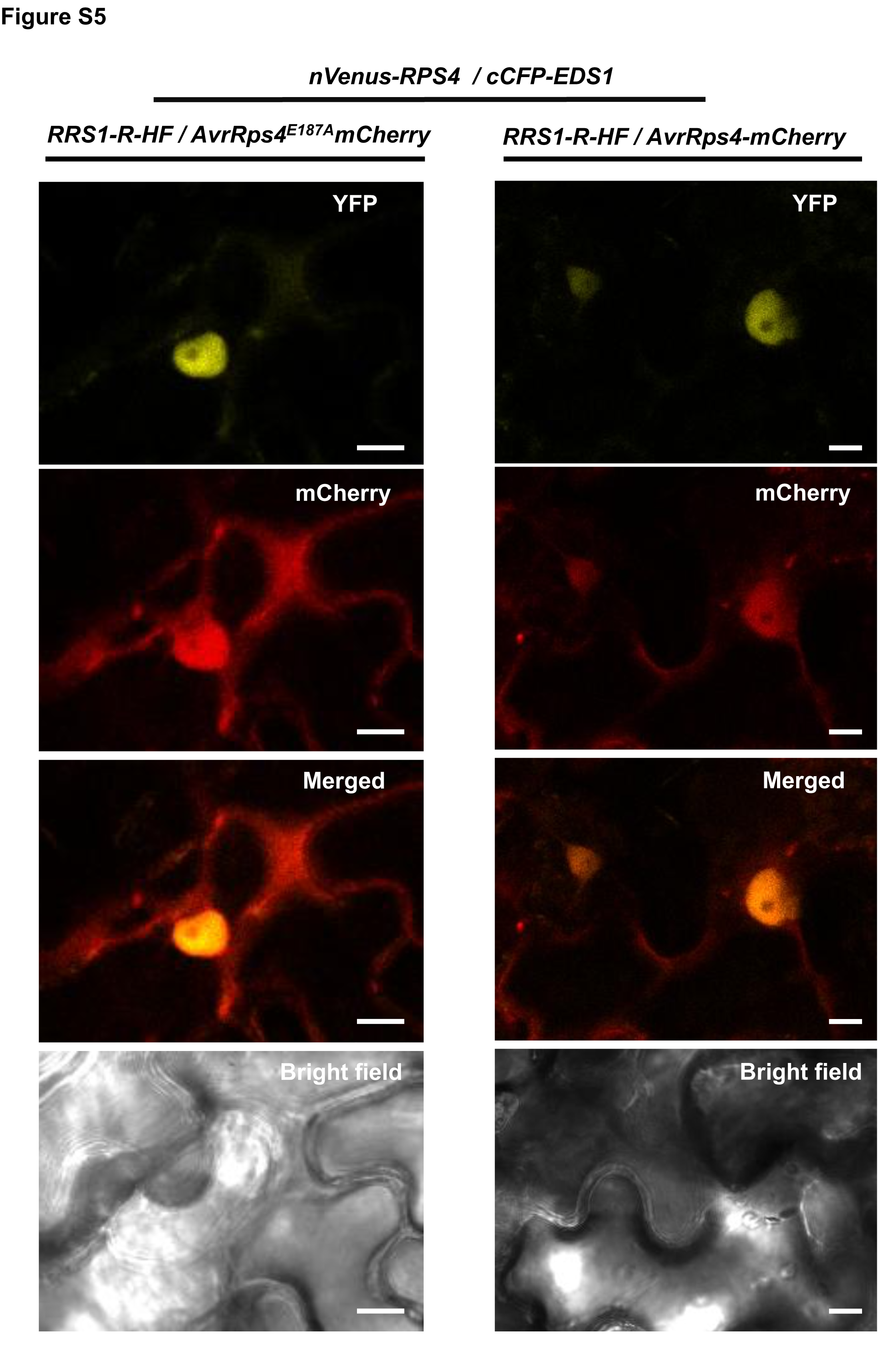

Supplement: S5 Fig — BiFC assays of RPS4/EDS1 association in the presence of RRS1 or both RRS1 and AvrRps4 or AvrRps4E187A. N. benthamiana leaves were co-infiltrated with nVenus-RPS4/nCFP-EDS1/RRS1-R-HF/AvrRps4E187A or nVenus-RPS4/nCFP-EDS1/RRS1-R-HF/AvrRps4-mCherry, reconstructed YFP signals (nVenus/nCFP combination) were observed at 2 dpi. In the presence of RRS1-R-HF, both cCFP-RPS4/nCFP-EDS1/AvrRps4E187A-mCherry and cCFP-RPS4/nCFP-EDS1/AvrRps4-mCherry complex provided similar nuclear YFP fluorescence. The experiment was repeated three times. Scale bar = 15 μm. (TIF) [file ppat.1006376.s005.tif]

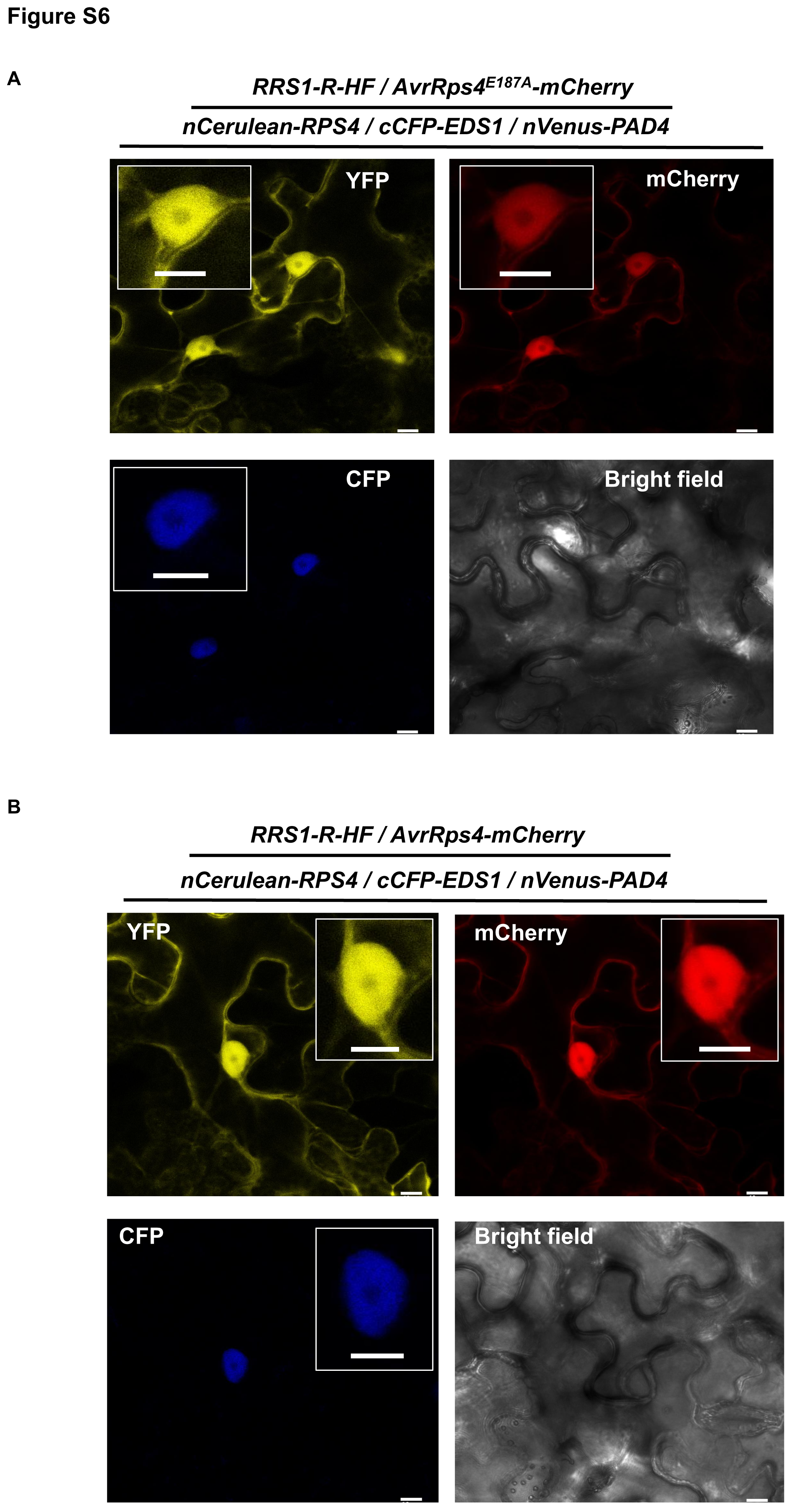

Supplement: S6 Fig — (A-B) Multi-color BiFC analysis between RRS1, RPS4, EDS1 and PAD4 in the presence or absence of AvrRps4. RRS1-HF, nCerulean-RPS4, cCFP-EDS1 and nVenus-PAD4 were transiently co-expressed with AvrRps4-E187A-mCherry or AvrRps4-mCherry, in N. benthamiana leaves. Co-expression of nCerulean-RPS4 and cCFP-EDS1 resulted in the reconstitution of CFP fluorescence within the nucleus. Co-expression of cCFP-EDS1 and nVenus-PAD4 reconstructed YFP fluorescence in both the nucleus and cytoplasm. No significant differences were observed in the presence of AvrRps4 or AvrRps4E187A-mCherry for both combinations. The experiment was repeated three times with similar results. Scale bar = 15 μm. (TIF) [file ppat.1006376.s006.tif]

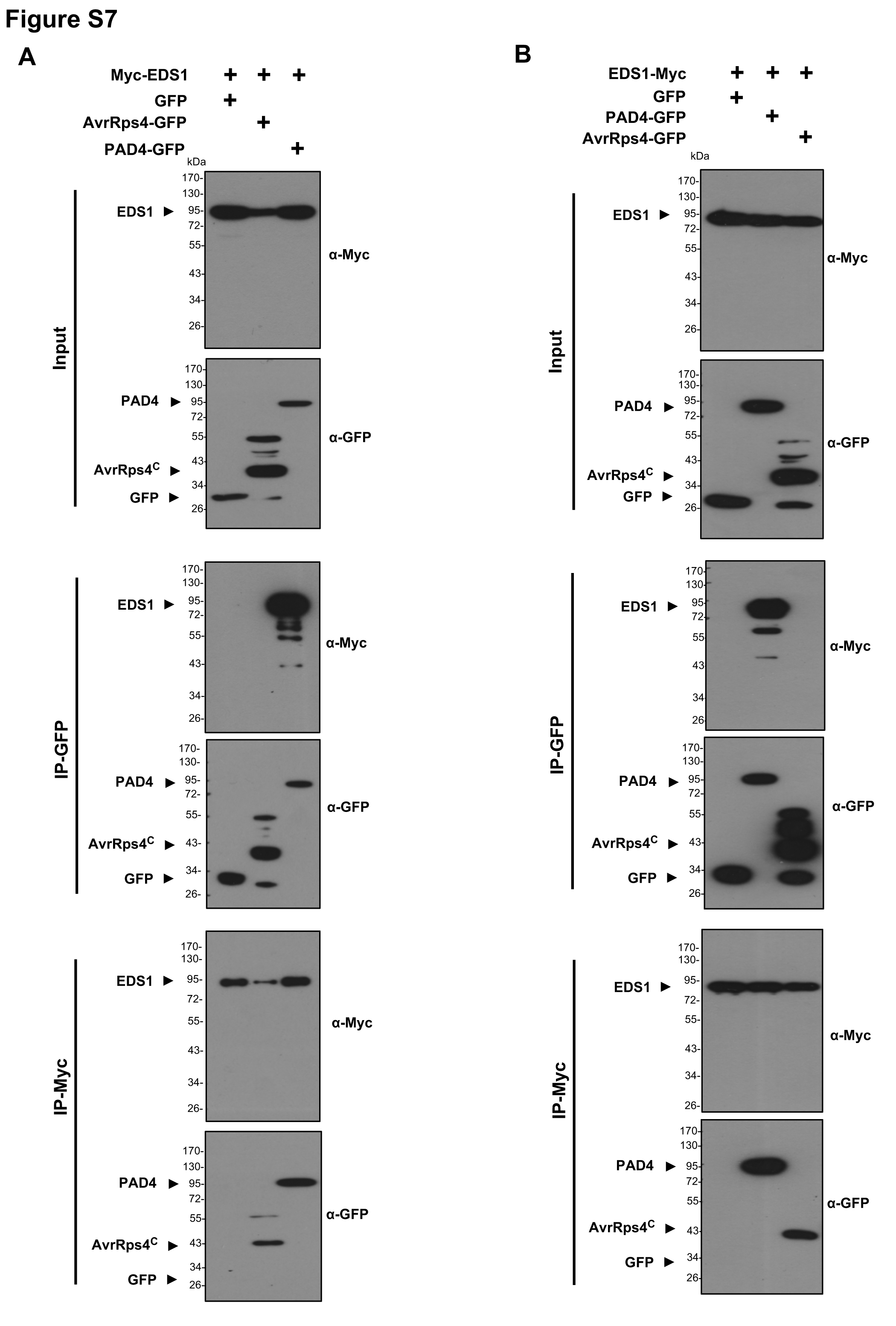

Supplement: S7 Fig — (A-B) Both N- and C-terminally Myc tagged EDS1 co-immunoprecipitate with AvrRps4 in planta. The 35S::Myc-EDS1 or the 35S::EDS1-Myc were co-infiltrated with the 35S::PAD4-GFP, 35S::AvrRps4-GFP or 35S::GFP in N. benthamiana leaves and samples were harvested at 2 dpi. Immunoprecipitations were performed using anti-GFP and anti-Myc agarose beads. Specific protein-protein interactions were detected by immunoblotting with the indicated antibodies. AvrRps4C represents processed AvrRps4C-terminus. The experiment was repeated three times with similar results. (TIF) [file ppat.1006376.s007.tif]

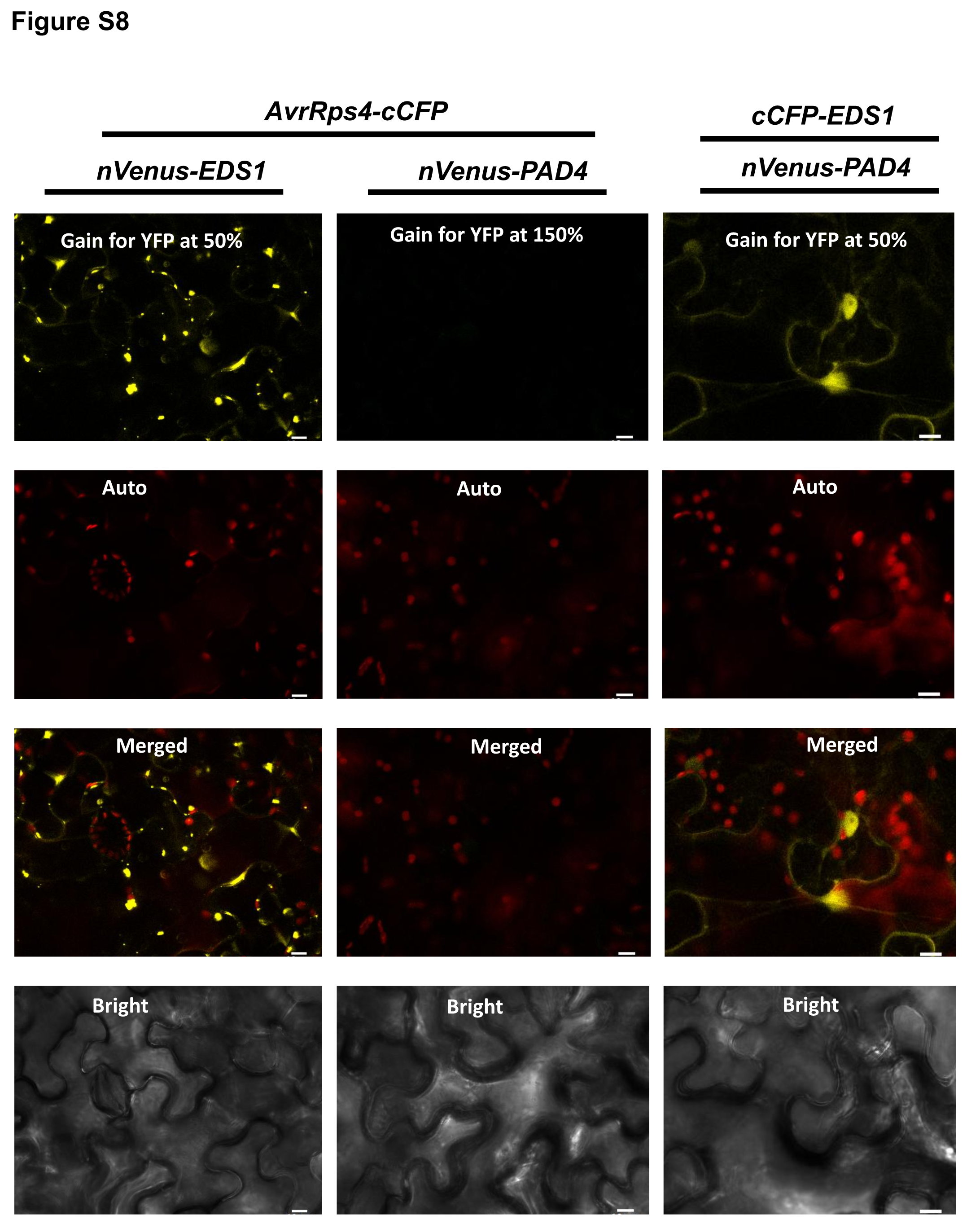

Supplement: S8 Fig — The AvrRps4-cCFP and nVenus EDS1 constructs were transiently co-expressed in N. benthamiana leaves. The combination of AvrRps4-cCFP with nVenus-PAD4 was used as a negative control. The functionality of nVenus-PAD4 construct was verified by co-expression with cCFP-EDS1. Red or blue fluorescence is the indicative of chloroplast auto-fluorescence. Reconstitution of yellow fluorescence protein (YFP) indicates protein-protein interactions. The experiment was repeated three times with similar results. Scale bar = 15 μm. (TIF) [file ppat.1006376.s008.tif]

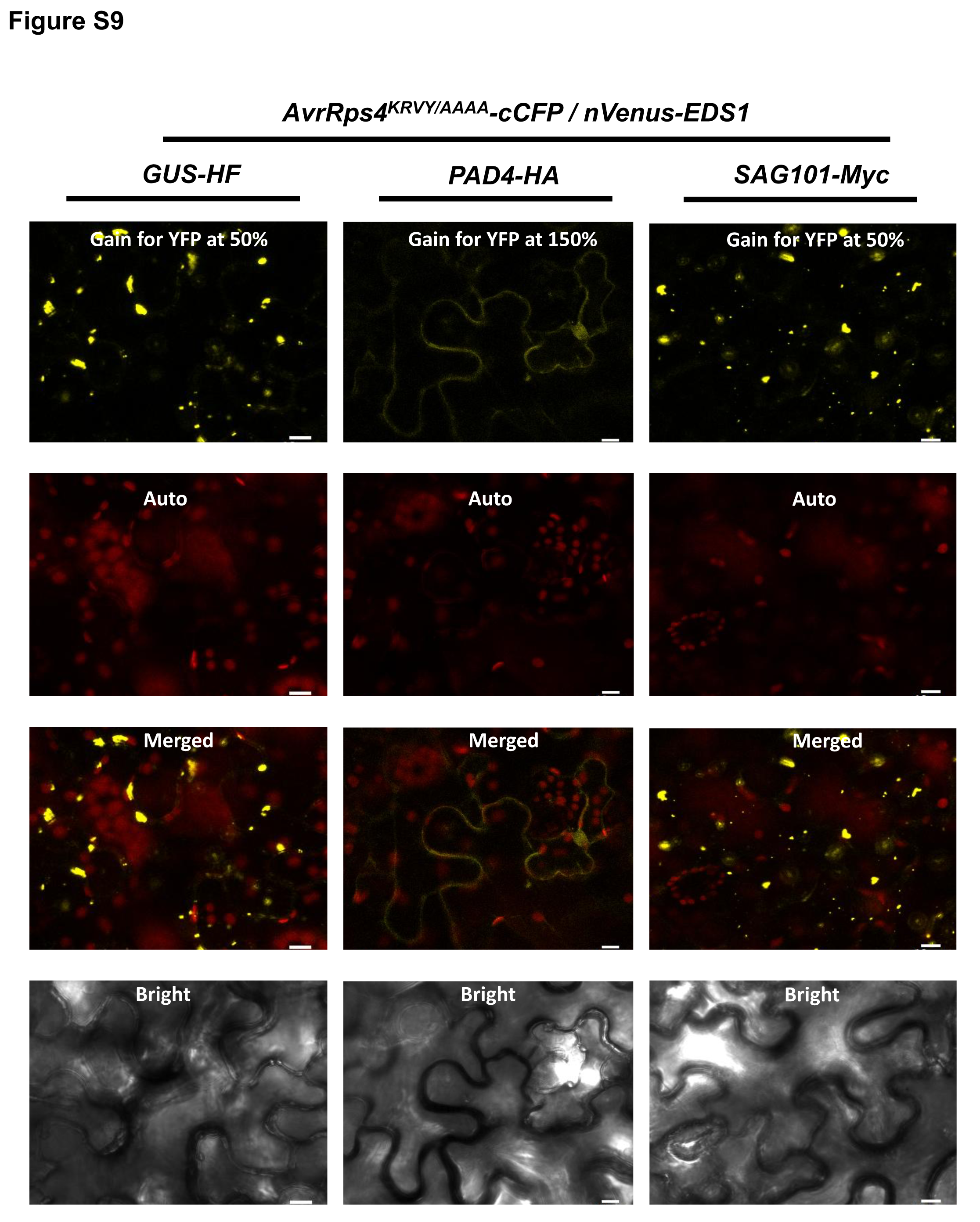

Supplement: S9 Fig — BiFC reveals that interaction between of EDS1 and AvrRps4KRVY/AAAA mutant forms cytoplasmic aggregations that are reduced in the presence of PAD4-HA but not in the presence of SAG101. BiFC assays were performed by co-expression of the indicated proteins in N. benthamiana. Scale bar = 15 μm. (TIF) [file ppat.1006376.s009.tif]
